# Flow Cytometric Detection of Biomarker Changes in CFDA‐SE‐Labelled Plasma Extracellular Vesicles Using a Rodent Pregnancy Model of Prenatal Diagnostics

**DOI:** 10.1002/jex2.70145

**Published:** 2026-05-05

**Authors:** Petra Adamova, Lesley Sloan, Andrew K. Powell, Iain M. Dykes

**Affiliations:** ^1^ Department of Pharmacy and Biomolecular Sciences Liverpool John Moores University Liverpool Merseyside UK; ^2^ Liverpool Centre for Cardiovascular Science Institute for Health Research Liverpool John Moores University Liverpool Merseyside UK

**Keywords:** blood biomarker, diagnostics, exosome, extracellular vesicle, flow cytometry, pregnancy, size‐exclusion chromatography

## Abstract

Circulating extracellular vesicles (EVs) offer a promising source of non‐invasive biomarkers for congenital disease diagnostics but robust assays for their detection are lacking. We used a rodent pregnancy model as a proxy for a prenatal diagnostics assay to test whether we could detect a difference in EV tetraspanin display between pregnant rats and non‐pregnant controls using standard flow cytometry. Carboxyfluorescein diacetate succinimidyl ester (CFDA‐SE) was used as a positive EV marker. CFDA‐SE positive particle count was linear but both size and concentration were below those measured by nanoparticle tracking analysis. Plasma proteins activated CFDA‐SE non‐specifically. Fractional analysis of eluates revealed that plasma protein is efficiently separated from EVs by size‐exclusion chromatography (SEC). We identify albumin within antibody storage buffers as the main source of false positives. Attempts to reduce background by quenching (trypan blue) and clean‐up (SEC) methods were ineffective. We then probed CFDA‐SE‐labelled EVs with antibodies to assay surface biomarker display using a quadrant method to measure double‐positive EVs. We observed a reduction in CD63 display in the pregnant condition but no change in CD9 or CD81. Using CD63 display level as a diagnostic test allowed detection of the pregnant condition with a sensitivity of 0.83 at specificity of 1 (AUC = 0.99).

## 1 Introduction

Liquid biopsy, the profiling of biomarkers present in blood, offers much promise for diagnostics. Although most tests currently focus on the detection of genetic variants in fragments of DNA present in circulation, there is also much interest in extracellular vesicles (EVs) (Ma et al. 2024). EVs are sub‐cellular membrane‐bound particles involved in endocrine signalling which transport cargoes between tissues. EVs therefore offer the opportunity to monitor dynamic changes associated with disease states.

Much work in EV diagnostics has focussed on the analysis of the microRNA cargo of EVs. EVs also display proteins within their limiting membranes, and these proteins may provide information about the EV. Some proteins indicate the origin of the EV, for example in man EVs released by the placenta often express placental alkaline phosphatase (gene name: *ALPP*) (Sarker et al. 2014). Other proteins may function in targeting blood EVs to specific tissues, for example tumour‐derived EVs have been shown to express unique combinations of integrins which mediate tropism towards specific organs during metastasis (Hoshino et al. 2015). The tetraspanins mediate membrane dynamics during the biogenesis of EVs and remain within the membrane of mature EVs (Toribio and Yanez‐Mo 2022). Although the tetraspanins are common markers of all EVs, there is evidence of a skewed distribution within EV subtypes: CD63 is enriched within endosome‐derived exosomes and less frequent in EVs derived by budding from the cell surface (ectosomes) while CD9 and CD81 exhibit the opposite distribution (Mathieu et al. 2021; Fordjour et al. 2022).

Flow cytometry is a useful method to analyse EVs. The lower limit of detection of a conventional flow cytometer is generally within the range of 300–500 nm (Botha et al. 2021). Small EVs, a category that includes exosomes and small ectosomes, range in size from 30 to 150 nm and therefore lie outside the normal detection range and fall within the noise. In recent years, a number of specialised high‐resolution machines have been developed for the detection of EVs, but these are not yet widely available for routine use. It would be preferable for clinical diagnostics to be able to detect EVs using a standard, inexpensive and widely available flow cytometer. In this study we used the BD Accuri C6+, an entry‐level system.

To separate small EVs from the instrument's noise, one of two strategies may be adopted. Firstly, EVs can be immunocaptured and tethered to a bead larger than 0.5 µm to bring the particle within the detected size range. This has two drawbacks: immunocapture of necessity selects for a subset of EVs expressing the epitope (Campos‐Silva et al. 2019) and therefore makes a priori assumptions on this. Secondly each bead will bind multiple EVs and therefore the assay gives only a semi‐quantitative approximation of EV numbers (Suárez et al. 2017). For these reasons a more satisfactory solution is to use a positive EV marker which will label the EV in situ and permit separation of the signal from noise. Non‐specific markers such as lipophilic dyes (e.g., DiI (Morales‐Kastresana et al. 2017), PKH26 (Morales‐Kastresana et al. 2017; Pužar Dominkuš et al. 2018), PKH67 (Libregts et al. 2018) and MemGlow/MemBright (Collot et al. 2019; Brealey et al. 2024) have been used, but these are limited by false positives (Libregts et al. 2018), including labelling of lipoproteins which may be present in plasma EV preparations (Brealey et al. 2024), and in some cases micelle formation (Morales‐Kastresana et al. 2017; Pužar Dominkuš et al. 2018).

**FIGURE 1:**
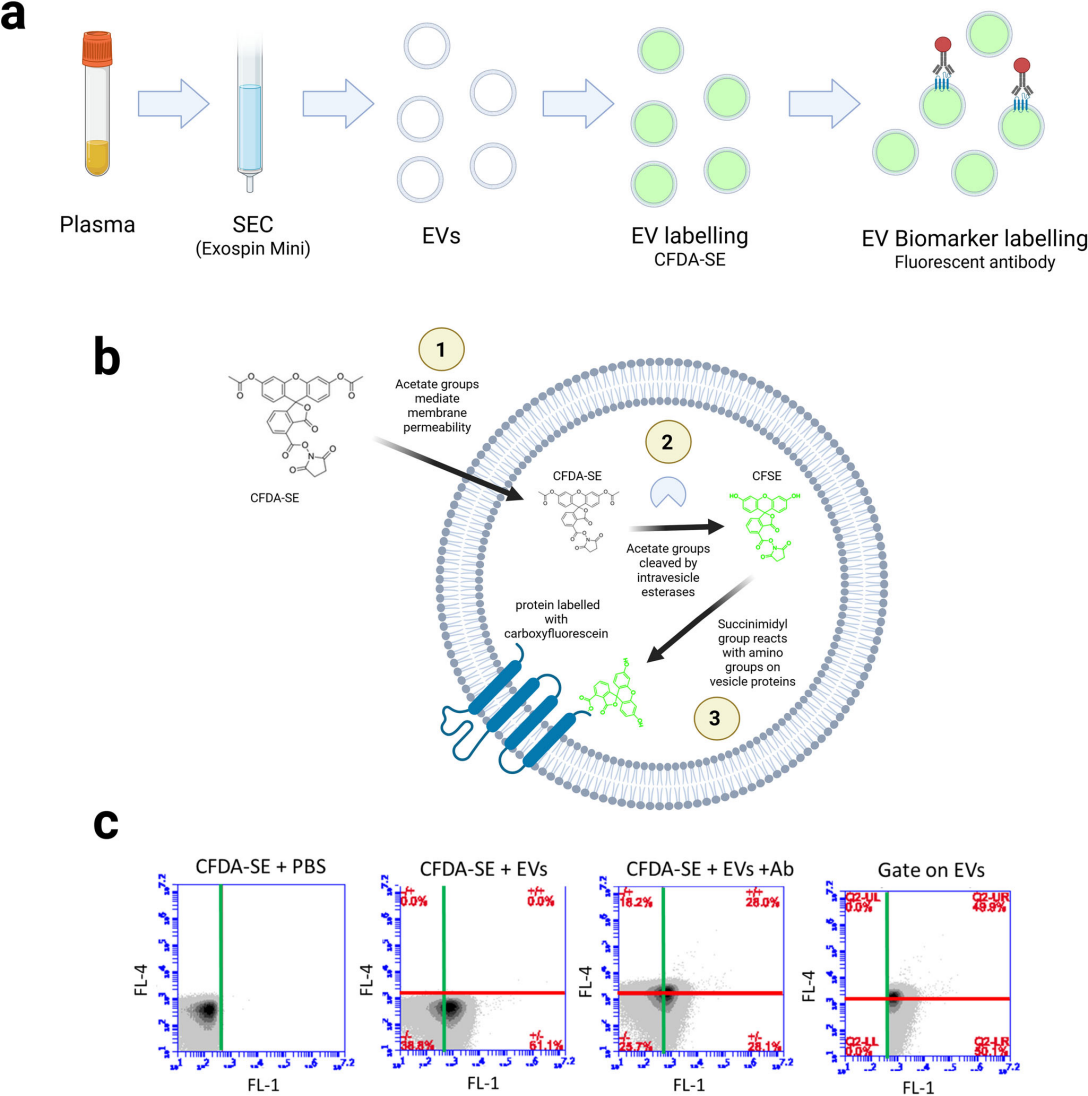
**Overview of experiment**. (a) EV‐enriched samples were prepared from rat plasma samples by Exospin Mini size exclusion chromatography. These samples were labelled with the fluorescent marker CFDA‐SE, which enables identification of EVs by green fluorescence. Antibodies carrying a red fluorescent tag were then used to identify subsets of EV displaying specific surface biomarkers. (b) CFDA‐SE is a non‐fluorescent derivative of the fluorophore carboxyfluorescein. (1) The acetate groups of CFDA‐SE allow it to cross the vesicle membrane. (2) Esterases inside the EV cleave the acetate groups to produce CFSE. This activates the green fluorescence of the dye. (3) The succinimidyl ester group reacts with the amino group of proteins within the vesicle. (c) Flow cytometry quadrant method to identify EV biomarkers. In the absence of EVs (CFDA‐SE+PBS), all particles exhibit low FL‐1 fluorescence and an FL‐1 threshold can be set (green line) to indicate the maximum fluorescent signal observed in the negative control. In the presence of EVs (CFDA‐SE+EVs), FL‐1 fluorescence increases. We defined any particle with a fluorescent signal above the threshold to be an EV. An FL‐4 threshold can be set (red line) to indicate the maximum fluorescent signal observed in the absence of antibody. In the presence of antibody FL‐4, fluorescence increases above threshold. A gate is applied to remove CFDA‐SE particles, leaving only CFDA‐SE+ particles which we define as EVs. The proportion of CFDA‐SE+ particles above the FL‐4 threshold is calculated. In this example, 49.9% of EVs express the marker. CFDA‐SE, carboxyfluorescein diacetate succinimidyl ester; EV, extracellular vesicle.

Perhaps a better solution is to use a label that is activated only within EVs and is therefore specific to them. To this end, dyes developed for live cell imaging and counting in flow cytometry assays can be repurposed as EV labels. This class of dye are lipophilic molecules that can cross the plasma or EV membrane and react with the amine groups of intracellular or intravesicular cargo proteins (Parish 1999). Critically, fluorescence must be induced or ‘switched on’ by hydrolysis of an inhibitory moiety attached to the fluorescent dye, this function is performed by esterases present within the vesicle. This ensures the signal is specific to membrane‐limited particles and reduces background due to similarly sized contaminants in the preparation. Examples include calcein AM (in which the fluorescent dye calcein is bound to acetoxymethyl) (Gray et al. 2015; De Rond et al. 2018), and carboxyfluorescein diacetate succinimidyl ester (CFDA‐SE, Figure 1) (Morales‐Kastresana et al. 2017; De Rond et al. 2018; Pospichalova et al. 2015; Ender et al. 2020; Maia et al. 2020; Mastoridis et al. 2018; Woud et al. 2022).

The aim of this work was to develop a robust method with which to assay EV surface biomarker display in EVs from blood using standard flow cytometry. Our starting material was plasma obtained from rats. The workflow involved purifying an EV‐enriched sample—preferably free of any contaminants present in blood—using size‐exclusion chromatography (SEC), fluorescent labelling of all EVs using CFDA‐SE and then labelling of a sub‐population expressing a given biomarker using fluorescent dye‐conjugated antibodies (Figure 1a). The chemistry of CFDA‐SE indicates that it should only fluoresce once inside an EV, and once inside that it should not be able to escape (Figure 1b). We defined an EV as a particle with fluorescence in the green (FL‐1) channel. By flow cytometry, we could then search for double‐positive particles, indicative of an EV displaying a given biomarker. The quadrant‐based gating strategy used is shown in Figure 1c.

The first part of this study describes our evaluation of CFDA‐SE labelling efficiency and specificity. We demonstrate that CFDA‐SE can be used as a fluorescent positive EV marker. Particle count is linear across a wide concentration range, indicating a lack of swarming, but both particle size and concentration values are below those calculated by nanoparticle tracking analysis (NTA). We identify a lack of specificity and demonstrate that CFDA‐SE may be activated outside vesicles by blood proteins such as albumin leading to false positives. We go on to investigate sources of albumin including both plasma itself and the storage buffers of antibodies used in the analysis. Our data suggest that the former is largely eliminated by SEC purification and that the latter is the greater source of background.

We investigate two methods to remove background: clean‐up and quenching. It has previously been reported that unbound CFDA‐SE may be removed from cell culture media EVs following labelling and that this may be an effective method to increase the sensitivity and specificity of the assay (Morales‐Kastresana et al. 2017). However, this assay was performed without antibody labelling of EVs, on ultracentrifuge‐purified EVs from cell culture media. We tested its applicability to our antibody‐labelled plasma EVs, identifying some problems that need to be addressed before the method can be widely adopted.

The trypan blue dye‐exclusion assay is commonly used in cell culture to count the proportion of live cells (Strober 2015). This assay is based on the principle that the negatively charged dye is unable to cross the plasma membrane of a healthy live cell (Strober 2001). Furthermore, trypan blue is known to quench the green emission of FITC or GFP dyes and combining these two properties allows researchers to separate intracellular from extracellular fluorescence for the study of uptake by endocytosis (Karpowicz et al. 2017), pinocytosis (Kerschbaum et al. 2021) and phagocytosis (Loike and Silverstein 1983). We hypothesised that trypan blue could be used to quench extra‐vesicle background CFDA‐SE fluorescence to improve the specificity of the signal. However, the results indicate that the EV membrane behaves as that of a dead cell and is permeable to the quenching dye.

In the second part of the work, we use the CFDA‐SE method to search for changes in the EV surface protein display of circulating EVs using our rat model of pregnancy as a surrogate for a prenatal diagnostics assay. EVs derived from the foetus may be present in maternal circulation [reviewed in (Adamova 2023)] and therefore analysis of such EVs offers the prospect of improved diagnostics for congenital disease. We show that the CFDA‐SE method can be used to compare biomarker expression between groups, provided that conditions are standardised. We identify a reduction in CD63 EV display in the pregnant condition and using this as a discriminator we could successfully detect pregnant rat plasma with a sensitivity of 0.83 at specificity of 1.

## 2 Methods

### 2.1 Experimental Design

We used a 6×6 design for the main biomarker analysis. This is sufficient for 80% power if the standard deviation is 55% of the mean difference between groups

### 2.2 Animal Work

The protocol for animal work described in this study was reviewed and approved by the Liverpool John Moores University Animal Welfare and Ethics Review Board in January 2020 (Ref: ID_PA/2023‐3). The blood samples used in this study have been previously described (Adamova et al. 2024), and the reader is referred to this paper for detailed methods regarding animal husbandry and blood collection. Briefly, a group of six pregnant rats (Gestation day E14.5) was compared to six control age‐matched non‐pregnant rats. Control and pregnant rats were housed in the same cages are therefore likely to be at the same stage in the oestrous cycle, although this was not verified. Blood samples were collected by post‐mortem cardiac puncture and centrifuged to obtain platelet‐free plasma.

### 2.3 EV Purification by Precipitation and Size‐Exclusion Chromatography (SEC)

An EV‐enriched sample was prepared from 100 µL of plasma using the Exospin Mini kit (Cell Guidance Systems, Cambridge, UK). Samples were pre‐cleared by spinning at 300 × *g* for 10 min to remove cell debris and then at 16,000 × *g* for 30 min to remove larger EVs (apoptotic bodies and larger microvesicles). The supernatant was then transferred to a new centrifuge tube and 50 µL of Exo‐spin Buffer was added in a 1:2 ratio. The tubes were mixed well by inverting and left to incubate at 4°C for 1 h. The mixture was then centrifuged at 16,000 × *g* for 1 h, the supernatant was aspirated and discarded, and the EV‐containing pellet was resuspended in 100 µL of PBS. An Exospin Mini SEC column was washed with 2 × 250 µL of PBS. The 100 µL sample was then added to the top of an Exospin SEC column and the flow‐through discarded. EVs were collected in the first 180 µL fraction (corresponding to Fractions 1–4 in our analysis of 45 µL fractions) by adding this volume of PBS to the column and collecting the flow through.

### 2.4 Fractional Analysis of Size‐Exclusion Chromatography Columns

Exospin Mini SEC columns were washed with PBS as above. One‐hundred microlitres of the test sample was then added to the top of the column and the flow‐through discarded. Forty‐five microlitres of PBS was then added to the column and the flow‐through collected (this is Fraction 1). This was repeated until a total of 20 fractions had been collected.

### 2.5 EV Storage

EV SEC preparations were aliquoted and stored in 20 µL aliquots at −80°C. Each aliquot was used for only one assay and was not refrozen. In our experience, EVs are more stable when concentrated.

### 2.6 CFDA‐SE Labelling

A stock solution of 40 µM CFDA‐SE (BD Biosciences) in DMSO was prepared and stored at −20°C.

All test solutions were prepared in PBS buffer in a final volume of 100 µL. For plate reader assays, solutions of either 10 µM or 19 µM CFDA‐SE in PBS were used as indicated in Section 3. Samples with EVs contain 20 µL of a 1:5 dilution of the EV SEC prep in PBS made fresh on the day. For all flow cytometry assays, a working solution of CFDA‐SE at 5 µM was made each day by adding 25 µL of 40 µM CFDA‐SE to 175 µL DMSO. Twenty microlitres of the 1:5 diluted EV SEC prep was used for each test sample and added to 20 µL of 5 µM CFDA‐SE and 60 µL of PBS, to give a final concentration of 1 µM CFDA‐SE and a final volume of 100 µL. Samples without EVs contained 20 µL of 5 µM CFDA‐SE and 80 µL of PBS. Samples were incubated at 37°C for 45 min before analysis.

### 2.7 CFDA‐SE and Antibody Dual Labelling

A list of antibodies used in this study, together with their isotype controls, is shown in Table 1. Twenty microlitres of a fresh 1:5 dilution of the EV SEC prep in PBS was used for each test sample, and labelling was performed in a final volume of 100 µL in PBS. All test solutions contained 1 µM CFDA‐SE. The labelling conditions were optimised for each antibody, and these are shown in Table 2. After labelling, each sample was diluted in PBS before analysis by flow cytometry.

**TABLE 1:** Antibodies and isotype controls used in this study.

Antibody	Class	Host	Conjugate	Test antibody	Isotype control
CD81	Monoclonal IgG1‐k	Hamster	APC	Biolegend 104910	Biolegend 400912
CD9	Monoclonal IgG1‐k	Mouse	APC	Biolegend 206504	Biolegend 400120
CD63‐B	Monoclonal IgG1	Mouse	AlexaFluor 647	Biorad MCAA4754A647	eBioscience S1‐4714‐81
CD63‐M	Monoclonal IgG1 (recombinant)	Human	APC	Miltenyi 130‐134‐122	Miltenyi 130‐113‐446

**TABLE 2:** Optimised staining conditions for dual labelling with antibody and CFDA‐SE.

Antibody	CFDA‐SE conc (µM)	Amount Antibody (µg)	Staining protocol
CD63‐B	1	0.5	Incubate at 37°C x 45 min in presence of antibody and CFDA‐SE. Dilute 1:20.
CD81	1	0.5	Incubate at 37°C x 30 min in presence CFDA‐SE only. Add antibody and incubate for 20 min at 4°C. Dilute 1:20.
CD63‐M	1	0.5	Incubate at 37°C x 30 min in presence CFDA‐SE only. Add antibody and incubate for 20 min at 4°C. Dilute 1:5.
CD9	1	0.04	Incubate at 37°C x 45 min in presence of antibody and CFDA‐SE. Dilute 1:20.

### 2.8 Detergent Treatment

Fifty microlitres of a fresh 1:5 dilution of the EV SEC prep in PBS was incubated with 50 µL of a 10% triton X100 solution for 30 min at room temperature before addition of CFDA‐SE. Samples were vortexed three times.

### 2.9 Flow Cytometry

The BD Accuri C6 Flow Cytometer (BD, New Jersey, USA) was used for all experiments. This machine is equipped with four channels named FL1 to FL4 (Table 3). Channels FL1–FL3 are excited using the same 488 nm laser and differ only in the emission filters used, while FL4 is excited using a 640 nm laser.

**TABLE 3:** Available fluorescent detection channels on the BD Accuri C6+.

FL channel	Excitation (nm)	Emission (nm)
FL‐1	488	533 ± 30
FL‐2	488	585 ± 40
FL‐3	488	670 long pass
FL‐4	640	675 ± 25

A wash cycle with water was performed at the start of all experiments, and a backflush was used between different samples to ensure there was no dye crossover. An Eppendorf containing 100 µL of each sample was placed under the sip. The fluidics were set to slow (flow rate 14 µL/min and core size at 10 µm ) in order to minimise any potential swarm effect. The threshold was set to 500 on forward scatter height (FSC‐H) and 1000 on side scatter height (SSC‐H) to minimise sample variation by attempting to exclude the collection of background noise. The endpoint used for all experiments was volumetric rather than event number because we considered this more accurate given that EVs lie within the size range of the system noise. A volume of 20 µL was set as a limit and read from each sample by the flow cytometer.

### 2.10 Calibration of the Flow Cytometer

Calibration was performed using 6‐Peak and 8‐Peak validation beads (Spherotech) following the manufacturer's instruction. We checked that 6 or 8 clearly defined peaks were present and that the coefficient of variation for the brightest peak on each was less than 5%. Calibration data are shown in Figure S1.

### 2.11 Determination of Particle Size by Flow Cytometry

To determine the size of EVs using flow cytometry, fluorescent polystyrene beads of known size (Table 4) were used to generate a calibration curve using the above flow settings. The bead populations were identified using the accompanying fluorescence. The mean and median FSC‐H values were recorded for each bead, log10 transformed and plotted on graphs. A linear relationship was observed between FSC‐H and particle size. A line of best fit was generated for each, and used to calculate the plasma EV size using both mean and median values.

**TABLE 4:** Polystyrene beads used in this study.

Size (nm)	Company	Catalogue code	Excitation (nm)	Emission (nm)	FL channel
30	Sigma–Aldrich	MFCD00131492	470	505	FL‐1
100	Sigma–Aldrich	MFCD00131492	475	540	FL‐2
500	Sigma–Aldrich	MFCD00131492	520	540	FL‐2

### 2.12 Plate Reader Assays

Plate reader analysis was performed using either a Spark multimode plate reader (Tecan, Mannedorf, Switzerland; with parameters: gain 100; number of flashes 30; integration time 40 µs) or a Clariostar Plus (BMG Labtech, Offenburg, Germany) with parameters: number of flashes 30; gain optimised for each run. Black plates were used for all runs.

### 2.13 Dilution Series Assay

Twenty microlitres of an undiluted SEC prep and 20 µL of a 1:5 dilution of the SEC prep were each stained in 1 µM CFDA‐SE in a 100 µL volume for 45 min at 37°C along with PBS and PBS + CFDA‐SE controls. Fifty microlitres of the undiluted sample was added to 50 µL of PBS to make a 1:2 dilution. The 1:5 sample was serially diluted by adding 50 to 50 µL of PBS to make dilutions at 1:10, 1:20, 1:40, 1:80, 1:160 and 1:320. All samples were analysed on the plate reader to obtain global fluorescence readings (FL‐1). Samples were then analysed on the flow cytometer. A threshold was set for FL‐1 using the PBS + CFDA‐SE control, and the percentage of particles above threshold calculated (% CFDA‐SE). All samples were tested in triplicates. Numbers were normalised to the maximum value (mean reading of undiluted samples).

### 2.14 Trypan Blue Assay

All components except trypan blue were combined in a 100 µL volume and incubated for 45 min at 37°C. The sample was then split into two aliquots of 50 µL. Fifty microlitres of PBS was added to the control samples, and 50 µL of 0.4% trypan blue (Mediatech, Manassas, USA) was added to the test samples.

### 2.15 Transmission Electron Microscopy (TEM)

Forty microlitres of the purified EV preparation was placed onto a piece of parafilm. A Formvar‐coated 200 mesh copper grid Cu (Agar Scientific, Rotherham, UK) was placed face down on the drop and particles allowed to adhere for 15 min at room temperature. To negatively stain particles, the grid was transferred to a 40 µL drop of 2% ammonium molybdate in water (pH adjusted to 6.85) for 20 min. Grids were washed by placing in a drop of distilled water, then air dried. Samples were examined with a Morgagni 268D Philips transmission electron microscope (FEI Morgagni, Field Electron and Ion Company, Oregon, USA), coupled with an Olympus Megaview III camera. Images were collected at x28,000 magnification.

### 2.16 Nanoparticle Tracking Analysis (NTA)

Ten microlitres of the purified EV preparation was diluted in 490 µL of PBS. NTA was performed using a Nanosight NS300 (Malvern Panalytical, Malvern, UK). Three technical replicates of each sample were performed.

### 2.17 Determination of Protein Concentration

A Nanodrop 2000 spectrophotometer (Agilent Technologies, Santa Clara, USA) was used to measure absorbance of a 2 µL sample at 280 nm.

### 2.18 Sensitivity and Specificity Calculations

The receiver operator characteristic (ROC) curves shown in Figure 8e–h are derived from the data shown in Figure 8a–d. Each data point was calculated by placing a threshold at a given percentage of CFDA‐SE particles expressing each biomarker and then counting the number of biological samples above or below that threshold. Mean values indicated whether a given biomarker gave a higher or lower signal in the pregnant condition. The true positive rate (TPR, or sensitivity) was then calculated as the number of pregnant rats above (or in the case of reduced biomarkers, below) the threshold divided by the total number of pregnant rats (*n* = 6). The false positive rate (FPR) was calculated as the number of non‐pregnant rats above or below the same threshold divided by the total number of non‐pregnant rats (n = 6). Specificity was calculated as 1 – FPR. The total area under the curve (AUC) was calculated as ∑(FPR2−FPR1)×(TPR2+TPR1)/2.

### 2.19 Graphs and Statistics

Graphs and statistics were plotted and performed using Excel, Graphpad Prism and Biorender.

### 2.20 Diagrams

Diagrams were prepared using Biorender.

### 2.21 Data Submission

We have submitted all relevant methods data related to our experiments to the EV‐TRACK knowledgebase (EV‐TRACK ID: EV240202) (Van Deun and Mestdagh 2017).

## 3 RESULTS

### 3.1 CFDA‐SE Labelling Identifies EVs

We first examined the fluorescent properties of our chosen EV label, CFDA‐SE (Figure S2). The predicted 488 nm/530 nm excitation/emission peaks for CFDA‐SE indicate that it should be detectable on the FL1 channel of the BD Accuri C6+ flow cytometer. We examined the full excitation/emission spectra of CFDA‐SE in PBS using a plate reader. CFDA‐SE exhibits a low level of fluorescence in solution before activation. We observed an excitation maxima of 504 nm, slightly higher than the expected value of 488 nm (Figure S2a), but indicating likely strong excitation of CFDA‐SE by the FL1 laser (Table 3). The emission maxima of 528 nm was close to the predicted value of 530 nm (Figure S2b) indicating likely strong emission within the FL1 emission filter range of 503–563 nm (Table 3).

**FIGURE 2:**
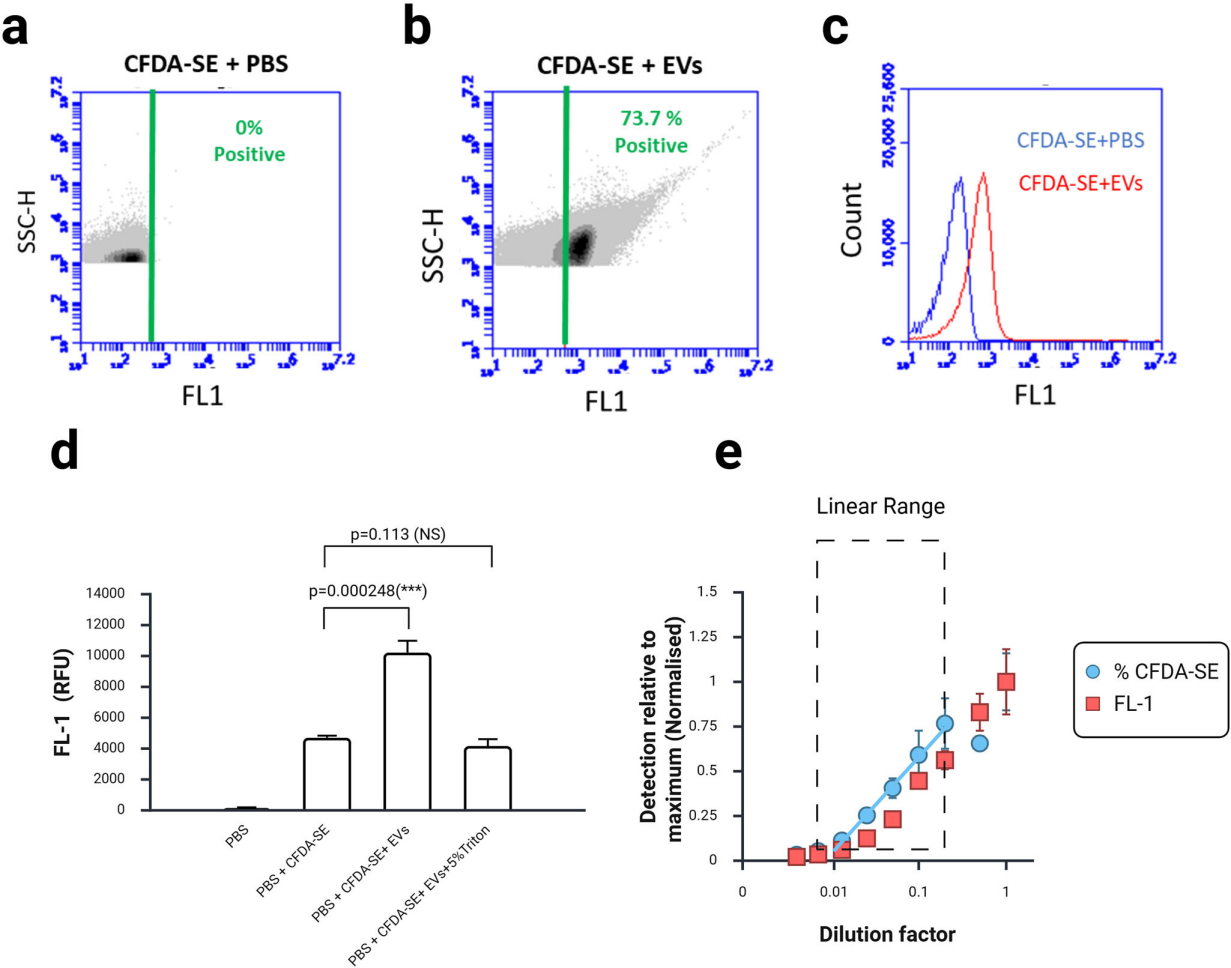
**CFDA‐SE fluorescence positively identifies EVs**. (a–c) BD Accuri C6+ flow cytometry data. (a) In the absence of EVs (CFDA‐SE+PBS) all particles exhibit low FL1 fluorescence. A threshold was set on the FL‐1 axis (green line) to indicate the maximum fluorescent signal observed in the negative control. (b) In the presence of EVs (CFDA‐SE+EVs), fluorescence increases. We defined any particle with a fluorescent signal above the threshold to be an EV. In this example, 73.7% of all particles in the preparation are EVs. (c) Histogram derived from data shown in a and b to indicate the increase in fluorescence in the presence of EVs (red) over the control (blue). (d) Plate reader data. There is a significant increase in global FL‐1 fluorescent intensity in the presence of EVs. Addition of the detergent Triton‐X100 to EVs before staining eliminates this effect. *n* = 3. *p* values indicate result of *t* test. All samples are normally distributed (Shapiro–Wilk test) and of equal variance (Levene test). (e) Graph to show dilution series of CFDA‐SE stained EV samples analysed both by plate reader (FL‐1 values) and by flow cytometry (% CFDA‐SE values). A linear relationship may be observed between the 1:5 (0.2) and 1:80 (0.0125) dilutions, but in very concentrated samples (1:2) the flow cytometer underestimates particle count, indicative of swarm detection. CFDA‐SE, carboxyfluorescein diacetate succinimidyl ester; EV, extracellular vesicle.

To demonstrate that CFDA‐SE labels EVs, we examined the change in fluorescence of CFDA‐SE in the presence of EVs both at the global level using the plate reader (Figure S2c, d) and at the particle level using the flow cytometer (Figure 2). In the absence of EVs (CFDA‐SE + PBS), all particles exhibited a low level of fluorescence (Figure 2a). We set a threshold at the maximum FL1 signal observed in this negative control (Figure 2a, green line). In the presence of EVs, the FL1 signal of many particles was raised above this threshold (Figure 2b, c).

To prove that this signal was due to the presence of EVs rather than a contaminant present in the sample, we used detergent to disrupt the vesicle membrane. Detergent treatment reduced the FL1 signal to that of negative controls (Figure 2d). Based on these data, we could therefore define an EV as a particle within our CFDA‐SE stained sample with an FL1 signal above threshold. This is the definition we use throughout this report.

To test whether the flow cytometer is accurately reporting single particles, we performed a dilution series and compared the level of global fluorescence determined by plate reader analysis to particle counts. We observed a linear relationship between particle count and dilution factor over a wide range (Figure 2e).

### 3.2 Specificity of CFDA‐SE Labelling and Sources of Background

To examine the specificity of CFDA‐SE labelling, we considered contaminants that may be present in plasma. Albumin is one of the most abundant proteins in blood, making up about half of the protein present. We therefore reasoned that this is a likely contaminant. We used bovine serum albumin (BSA) as a proxy for rat serum albumin.

**FIGURE 3:**
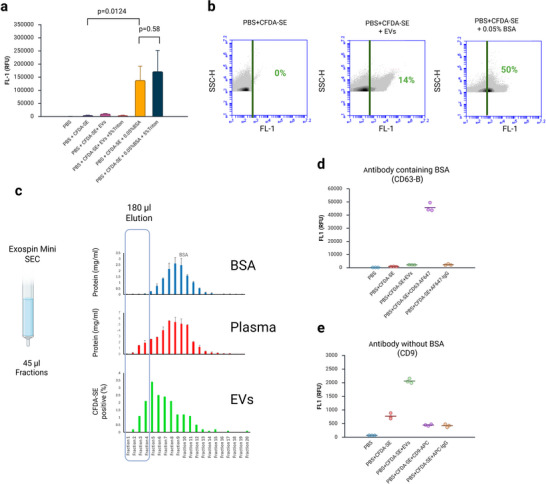
**BSA activates CFDA‐SE and results in false positives**. (a) A solution of 0.05% BSA strongly activates CFDA‐SE, and this signal is not eliminated by detergent treatment. Plate reader data. (b) Flow cytometer data. 0.05% BSA activates CFDA‐SE resulting in fluorescent particles above threshold (right panel) which are indistinguishable from CFDA‐SE labelled EVs (middle panel). (c) Exospin SEC columns efficiently remove protein from EV samples. Graphs show analysis of 20 × 45 µL samples collected from the SEC column. In the top row 100 µL of 1% BSA was put through the column and the protein concentration of each measured by Nanodrop analysis. In the middle row the same experiment was repeated with 100 µL of rat plasma. In the lower row, 100 µL of rat plasma was purified using an SEC column and the first 180 µL elution collected. One‐hundred microlitres of this was put through a second SEC column, fractions were stained with CFDA‐SE and the proportion of particles above threshold measured. (d) The CD63 (B) antibody, which contains 1% BSA, activates CFDA‐SE in the absence of EVs. The isotype control IgG‐AF647 does not have a large effect. Plate reader data. (e) The antibody CD9, which does not contain BSA, does not activate CFDA‐SE in the absence of EVs. Plate reader data. BSA, bovine serum albumin; CFDA‐SE, carboxyfluorescein diacetate succinimidyl ester; EV, extracellular vesicle; SEC, size‐exclusion chromatography.

We found that BSA strongly activates CFDA‐SE fluorescence, detected as a large FL‐1 signal (Figure 3a). This fluorescence is not eliminated by detergent treatment (Figure 3a). This indicates that CFDA‐SE may be reacting with any proteins present in our EV‐enriched sample. Furthermore, the presence of BSA results in the presence of fluorescent particles above threshold in our flow cytometer assay, which are indistinguishable from CFDA‐SE labelled EVs (Figure 3b).

We therefore investigated whether BSA or other proteins may be present in our samples, leading to false positives. The most obvious source of protein is from the original plasma sample, in which albumin is abundant. We enrich EVs from plasma using the Exospin Mini SEC column. We performed a series of tests to investigate whether the SEC column can separate EVs from blood proteins. We first ran a sample of BSA through the column and determined in which fraction the protein elutes. Our EV‐enriched sample is derived from a 180 µL elution applied to the column immediately after sample loading. To gain better resolution, we performed this assay using a series of 20 × 45 µL elutions (thus Fractions 1–4 correspond to the 180 µL fraction normally collected). The results showed that the majority of protein eluted in Fractions 6–13 (Figure 3c). There was very little protein in Fractions 1–4.

Next, we investigated which fractions EVs elute in. To perform this assay, we used a sample of plasma EVs that had already been purified using an SEC column and collected in Fractions 1–4. From the above result, we were now confident that this sample was largely free of protein. We ran this sample through a second SEC column, stained eluted fractions with CFDA‐SE and compared particle counts using the flow cytometer. These data revealed that EVs elute in Fractions 3–11, with a peak in Fraction 5 (Figure 3c).

Finally, we ran a sample of plasma through the column and measured protein content. This sample contains both EVs and albumin, both of which will be detected in this assay. As expected, protein is detected in a wider range than either of the previous samples, being present in Fractions 3–13.

A second potential source of background in our assay is the antibodies themselves. BSA is often present in antibody storage buffers. The storage buffer composition of antibodies used in this study is shown in Table S1. The CD63 (B) antibody contains 1% BSA, the CD9 and CD81 antibodies do not contain BSA, while the CD63 (M) antibody contains an unspecified protein stabiliser. We tested whether these antibodies can activate CFDA‐SE in the absence of EVs. The results showed that an antibody containing BSA (CD63 (B)) does activate CFDA‐SE (Figure 3d, Figure S3) while an antibody that does not (CD9) has no background signal (Figure 3e). The CD63 (M) antibody showed a level of background, while the CD81 had none (Figure S3).

### 3.3 Background Reduction: SEC Cleanup

**FIGURE 4:**
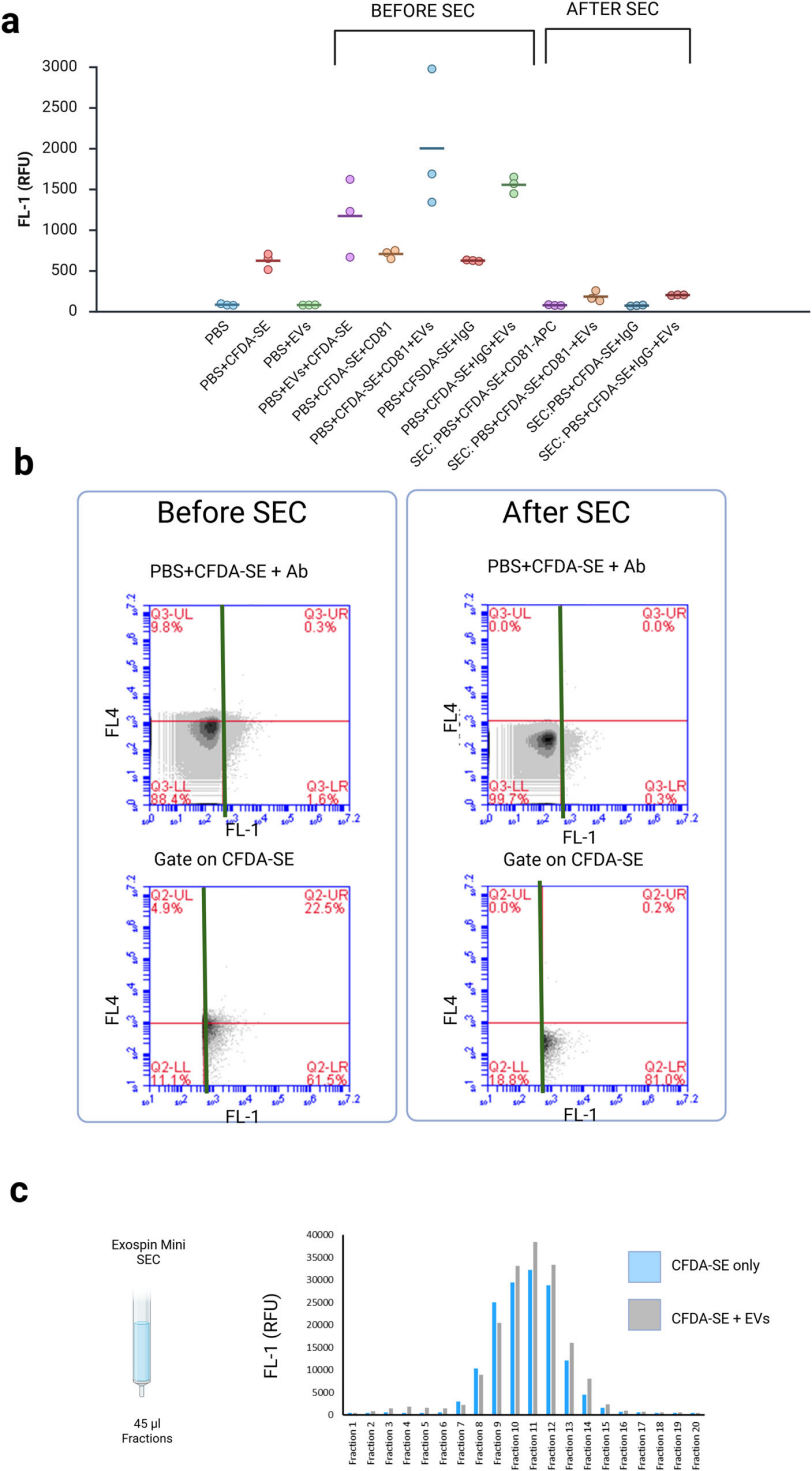
**Size exclusion chromatography inefficiently recovers EVs labelled with CFDA‐SE and antibody**. (a) Plate reader data. Before SEC, samples containing EVs and CFDA‐SE show an FL‐1 signal above the PBS+CFDA‐SE control. Following SEC purification and recovery of the first 180 µL fraction the FL‐1 signal drops below the PBS+CFDA‐SE control. (b) Flow cytometry data showing tha PBS+CFDA‐SE+EVs+CD81 sample. Before SEC, 22.5% of CFDA‐SE positive samples are positive for FL‐4. Following SEC purification and recovery of the first 180 µL fraction only 0.2% of these particle are above the FL‐4 threshold. (c) Plate reader data. CFDA‐SE in PBS (blue) or CFDA‐SE in PBS with EVs (grey) were purified using the SEC column and 20×45 µL fractions collected. CFDA‐SE labelled EVs appear to elute later than unlabelled (Figure 3c) and to elute in the same fractions as unbound CFDA‐SE. CFDA‐SE, carboxyfluorescein diacetate succinimidyl ester; EV, extracellular vesicle; SEC, size‐exclusion chromatography.

We next asked whether it might be possible to reduce the background signal introduced by antibodies that have BSA in their storage buffer. We first looked at the possibility of removing unbound CFDA‐SE by ‘cleaning up’ the sample using an SEC column. For this test, we used the CD81 antibody, which does not contain BSA, in order to avoid any ambiguity in the detection of EVs. We examined four samples containing either the CD81 or the isotype control with and without EVs. Each sample was analysed before and after putting through an SEC column (collecting the 180 µL fraction). The results showed that FL‐1 signal of EVs labelled with both CFDA‐SE and CD81 was reduced from a mean value of 1998 before SEC to 180 after SEC, a level close to that of PBS alone (80; Figure 4a). Furthermore, the ratio of the FL‐1 signal between samples with and without EVs (i.e., the signal:noise) was reduced from 2.85 to 2.44 after SEC. Before SEC purification, we determined that 22.5% of EVs express CD81 using our quadrant method (Figures 1c, 4b). After SEC purification, analysis of the same sample revealed a large reduction in both FL‐1 and FL‐4 positive particles, with very few detectable double positive particles (Figure 4b). Thus, we concluded that samples cannot be cleaned up after staining.

To investigate what might be happening we ran a sample of unbound CFDA‐SE dye through the SEC column, and used the autofluorescence resulting from spontaneously hydrolysed dye to measure the FL‐1 signal. This analysis revealed that free CFDA‐SE dye elutes in Fractions 8–14 (Figure 4c, blue bars). We then ran a sample of CFDA‐SE‐stained EVs through the column signal and measured the FL‐1 signal of eluted fractions. We observed that most of the FL‐1 signal occurred in the same Fractions 8–14 with only limited detectable signal in Fractions 2–6 (Figure 4c, grey bars). These data suggest that the movement of labelled EVs through the SEC column is impeded, resulting in elution in the same fractions as free dye.

### 3.4 Background Reduction: Trypan Blue Quenching

We then asked whether it is possible to quench the background fluorescence to improve the EV fluorescent signal using trypan blue.

**FIGURE 5:**
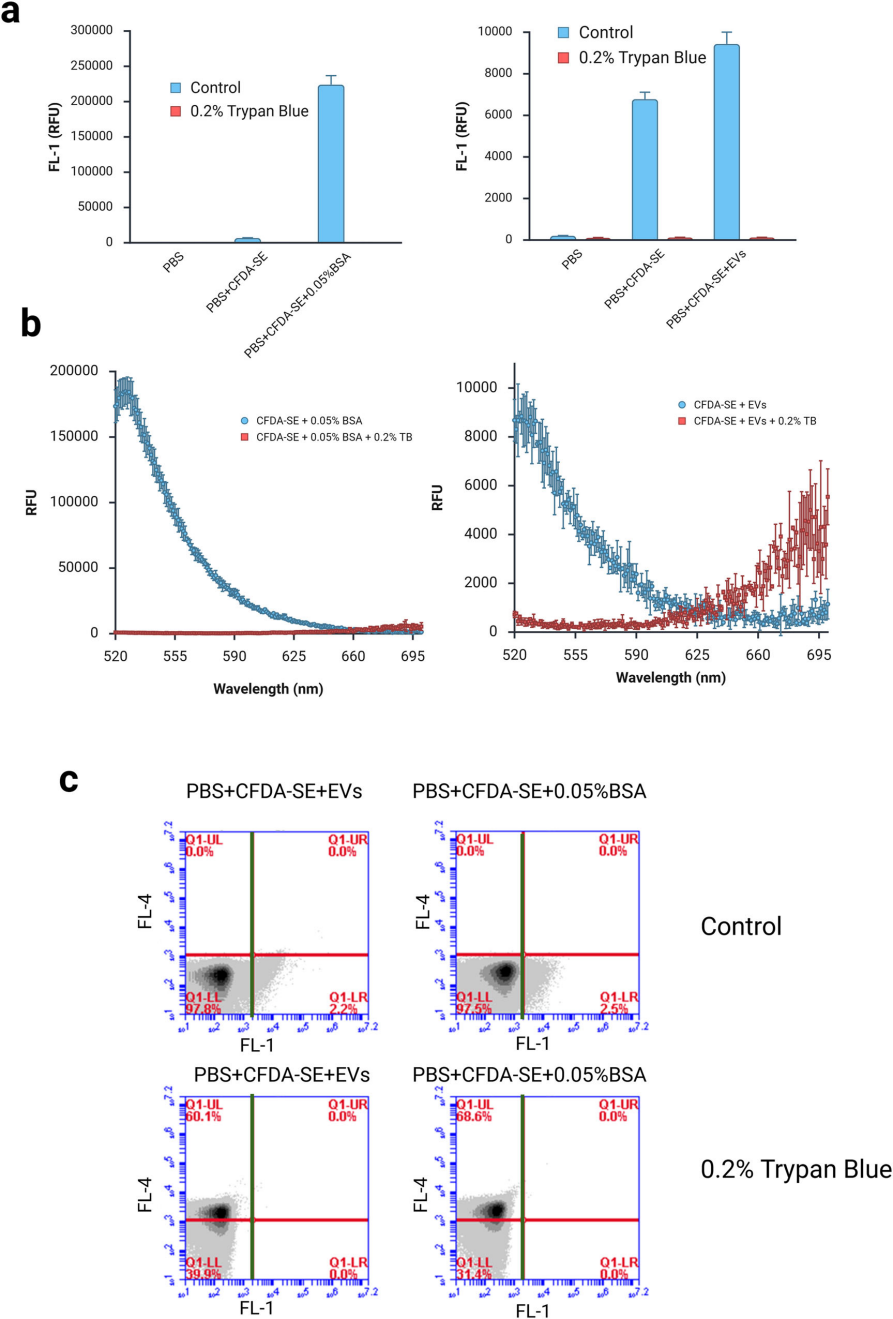
**Trypan blue is unsuitable to quench background fluorescence**. (a) 0.2% trypan blue was added to CFDA‐SE labelled EVs (left) and CFDA‐SE labelled BSA (right). These were compared to control labelled samples without trypan blue. Graphs of FL‐1 fluorescence intensity shows that trypan blue quenches both BSA and EV signal. Plate reader data. (b) Plate reader data. Emission scan with excitation at 488 nm. CFDA‐SE labelled BSA (left) and EVs (right). Blue points control, red points 0.2% trypan blue. (c) Flow cytometer data of the same samples showing FL4/FL1 quadrants. BSA, bovine serum albumin; CFDA‐SE, carboxyfluorescein diacetate succinimidyl ester; EV, extracellular vesicle.

The data showed that trypan blue eliminates FL‐1 fluorescence of both labelled BSA and labelled EVs (Figure 5a). In both cases, fluorescence is reduced to the level of PBS. We performed an emission scan with excitation at 488 nm. This revealed that not only does trypan blue eliminate emission within the FL‐1 detection range (533 ± 30 nm), but it also results in emission within the FL‐4 detection range (675 ± 25 nm, Figure 5b). Particle level analysis on the flow cytometer revealed that all particles are below the FL‐1 threshold in the presence of trypan blue while a population of FL‐4 particles appears (Figure 5c). Thus, we concluded that trypan blue is unsuitable for quenching the background signal.

### 3.5 Determination of Particle Size and Concentration

We next examined the ability of the CFDA‐SE flow cytometry method to assess particle size and concentration in a biological sample. For this, we used our rat pregnancy model to compare pregnant and non‐pregnant conditions.

**FIGURE 6:**
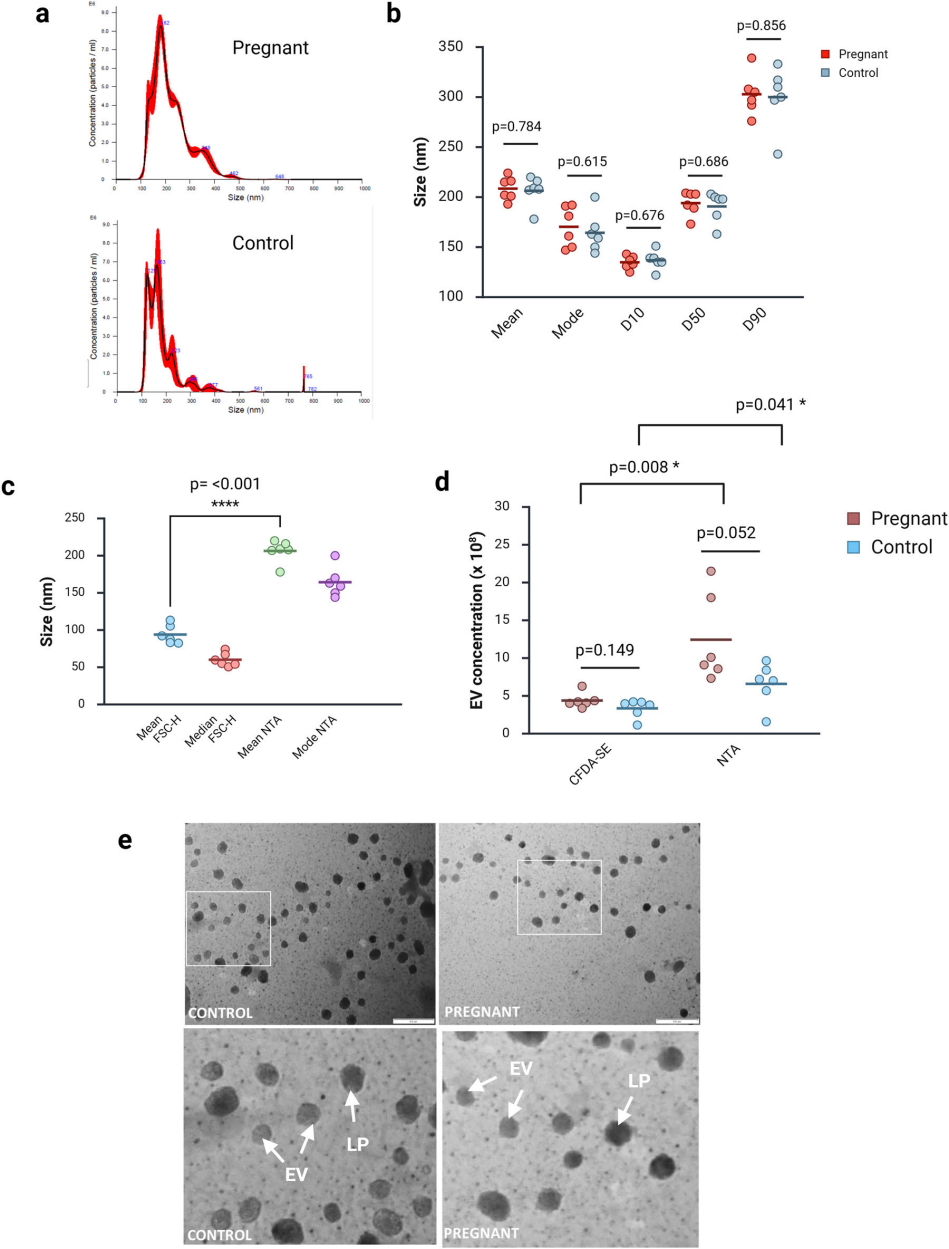
**Measurement of EV size distribution, concentration and morphology**. (a) Representative plots of nanoparticle tracking analysis (NTA) data for one pregnant and one control sample. Full dataset shown in supplementary data. (b) Summary of NTA size distribution data. A number of variables are shown here to capture the complex size distribution pattern. D10, D50 and D90 indicate the minimum size, which includes 10%, 50% or 90% of the data points, respectively. *p* values indicate result of *t* test. Samples are normally distributed and of equal variance. No difference is seen between the pregnant and control groups in any measure of EV size distribution. (c) Validity of CFDA‐SE staining to calculate EV size. Data show control group only. Size of CFDA‐SE‐stained particles was estimated by comparing either the mean or median forward scatter height (FSC‐H) to a calibration curve generated using polystyrene beads of known size. This is shown alongside values calculated using NTA mean and mode values. Using CFDA‐SE significantly underestimates particle size (*t* test, *p* < 0.0001). (d) Validity of CFDA‐SE staining to calculate EV concentration. Particle counts obtained from CFDA‐SE staining are compared to values calculated using NTA. NTA values are significantly higher than those calculated using the CFDA‐SE method. No significant difference is seen between pregnant and control groups with either method, although the NTA figure is close to the *p* = 0.05 threshold of significance. *p* values indicate result of *t* test (preg NTA vs. control NTA; preg NTA vs. preg CFDA‐SE) or Mann–Whitney *U* test (preg CFDA‐SE vs. control CFDA‐SE; control CFDA‐SE vs. control NTA). (e) TEM images of negative stained samples (28,000x). Left images show a control sample while right images show a pregnant. Blown‐up regions of the same samples are shown below, and these are indicated by the white boxes in the low power images. EVs: putative EVs, which have a lighter appearance, a visible outer membrane and a ‘squashed football’ shape. LP: large particles, putative LDL or other contaminants, which have a darker appearance without an obvious membrane. CFDA‐SE, carboxyfluorescein diacetate succinimidyl ester; EV, extracellular vesicle.

We first assessed the particle size within our samples using NTA (Figure 6a, b; Figure S4). This revealed a complex distribution, with a number of populations of different sized vesicles within our samples. This distribution cannot be easily captured in a single measure, and so we provide five different measures in Figure 6b. As an example, the mean particle size was 208.5 ± 11.6 nm in the pregnant group and 206.3 ± 14.8 nm in the non‐pregnant group. No difference between the groups was detected in any measure.

We measured particle size of CFDA‐SE stained EVs by comparing FSC‐H value values to beads of known sizes. Calculated mean sizes were 63.5 ± 8.0 nm for the pregnant group and 60.1 ± 8.9 nm for the non‐pregnant group. We did not detect a significant difference between pregnant and control groups. The values calculated using this method are significantly lower than those calculated by NTA (Figure 6c).

Next, we calculated EV concentration using both methods. The mean EV concentration calculated by NTA was 12.4 ± 5.8 × 10^8^ particles per mL plasma for the pregnant group and 6.6 ± 2.6 × 10^8^ particles per mL plasma for the pregnant group. This difference was just above the threshold for significance (*p* = 0.052). The mean EV concentration calculated by counting fluorescent particles above threshold following CFDA‐SE staining was 4.38 × 10^8^ ± 0.99 × 10^8^ particles per mL plasma for the pregnant group and 3.35 × 10^8^ ± 1.19 × 10^8^ particles per mL plasma for the control group, which was also not significant. The values calculated using this method are also significantly lower than those calculated by NTA (Figure 6d).

To examine the morphology of particles present in our samples we used TEM. This revealed the presence of particles with the classical morphology of an EV, being round, light in colour with an obvious membrane and often a ‘squashed football’ morphology (Figure 6e). These particles would be expected to stain with CFDA‐SE due to the presence of a membrane. We also noted the presence of a second type of particle (Figure 4d; purple arrows). These latter particles were generally larger than the former, more irregular in shape, darker in colour and without a clearly defined membrane. There is no positive EV marker in a TEM image, and therefore these preparations will contain any particles within the size range of the SEC column (30–250 nm), including both EVs and any non‐EV particles that may be present. LDL particles, which are common in blood, range in size from 100 to 500 nm and appear as dark, electron‐dense particles in TEM images (Sódar et al. 2016). Therefore, we hypothesised that the larger darker particles visible in our preparations may be LDL. These particles should not stain with CFDA‐SE due to the absence of a membrane.

### 3.6 Antibody Optimisation

Our biomarker assay depends on being able to compare the proportion of EVs stained with an antibody between groups. Before doing this, we looked at optimising the staining conditions.

We began by verifying the excitation/emission spectra for each antibody fluorophore (Figure S5). The observed excitation maxima was within 2 nm of the expected for all three fluorophores while the observed emission maxima was within 3–9 nm from expected but in all cases was within the range picked up by the FL‐4 filter.

**FIGURE 7:**
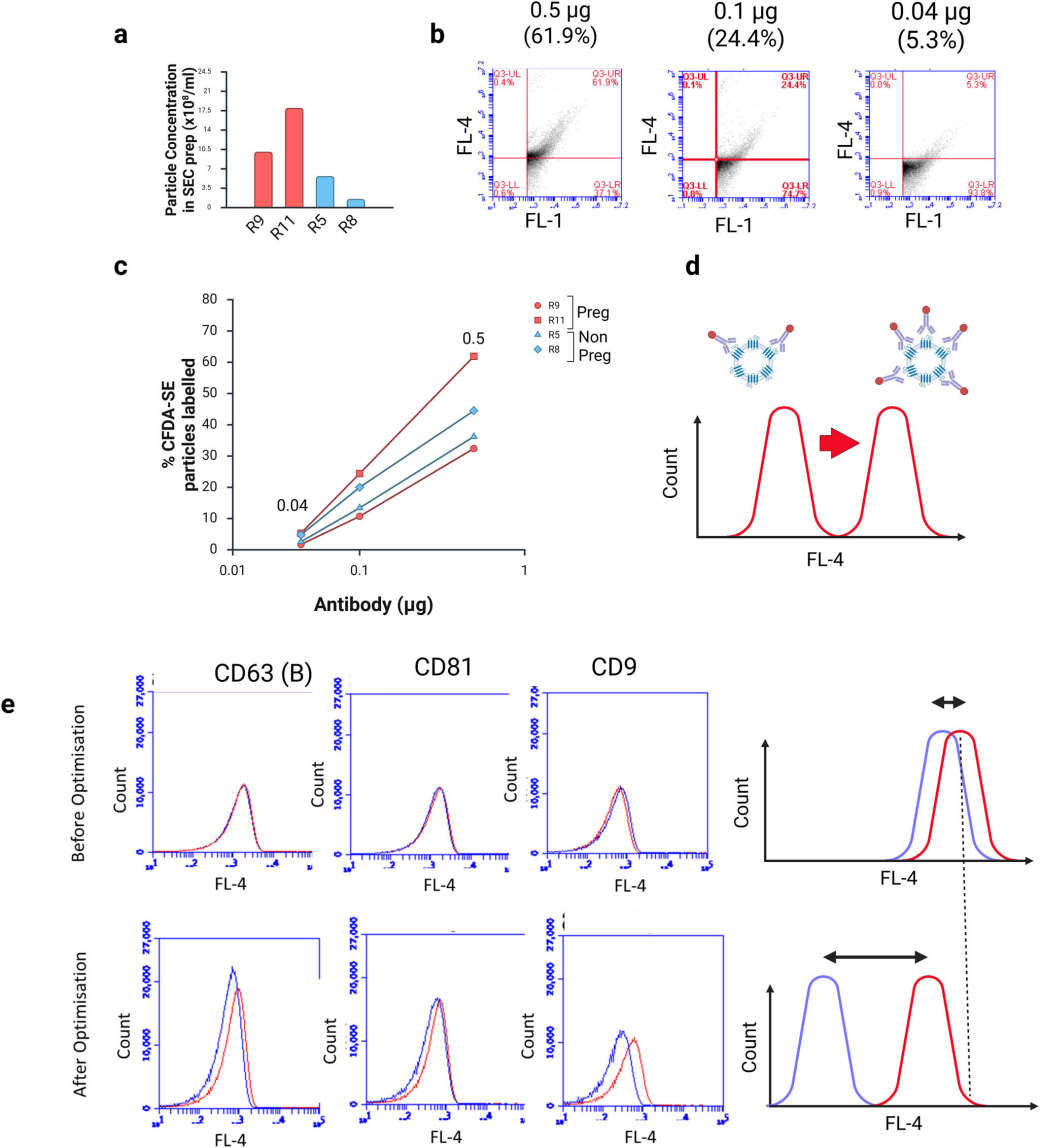
**Effect of antibody concentrations and optimisation of staining conditions**. (a–d) The CD9‐APC antibody was tested at three different concentrations (0.5, 0.1 and 0.04 µg in a 100 µL staining reaction). Four rat plasma SEC preps were tested including two pregnant and two non‐pregnant. (a) Graph shows relative number of EVs within each sample (NTA data). (b) Example flow cytometry data for rat R11. (c) Graph to show the proportion of CFDA‐SE positive particles labelled with the antibody. (d) Diagram to illustrate results. Each EV contains multiple binding sites for each antibody. Because the antibody is in limited supply, more antibody molecules will bind to each EV at higher antibody concentrations. This results in the EV appearing brighter when assayed by flow cytometry. As a result more EVs are observed above the FL‐4 threshold. (e) Optimisation of staining conditions for each antibody. Histograms to show distribution of FL‐4 intensity within the CFDA‐SE positive EV population. Before optimisation (upper images), the traces for the antibody (red) and isotype control (blue) overlap due to non‐specific binding of isotype. After optimisation (lower images), there is a clear separation between antibody and isotype. This is shown in the diagrams to the right, in which separation is exaggerated for illustrative purposes. CFDA‐SE, carboxyfluorescein diacetate succinimidyl ester; EV, extracellular vesicle; NTA, nanoparticle tracking analysis; SEC, size‐exclusion chromatography.

We then investigated the effect of antibody concentration within the staining reaction on the proportion of EVs shown to be double positive. To do this, we chose four samples with a range of EV concentration (as determined by NTA; Figure 7a), including both pregnant and non‐pregnant samples. Three different amounts of antibody were tested ranging from 0.004 to 0.5 µg. We again chose an antibody without BSA (CD9). Using our quadrant method (Figure 1c), we determined the number of double‐positive particles in each. The results showed that increasing antibody concentration increased the mean FL‐4 fluorescence of the particle population (Figure 7b), and this resulted in increasing numbers of double‐positive particles. There was a linear relationship between the log of antibody amount and particle count (Figure 7c). These results suggest that each EV has multiple binding sites for the antibody and that because the antibody is in limited supply, the number of antibody molecules bound to each EV increases as concentration increases, resulting in increased FL‐4 fluorescence (Figure 7d).

We used matched isotype controls to investigate the specificity of antibody binding. We found that the distribution histograms of FL‐4 fluorescence for both the antibody and isotype overlapped, especially when the antibody was used at a high concentration (Figure 7e). This indicated non‐specific binding of the antibody. To reduce this background we optimised staining conditions by reducing antibody concentrations and reducing the temperature until we saw a separation between the antibody and isotype, indicating that more antibody was binding to each EV than isotype (Figure 7e). Table 2 shows optimised staining conditions.

### 3.7 Profiling of Changes in Tetraspanin EV Expression in a Rat Pregnancy Model

Finally, we validated our CFDA‐SE flow cytometry method by profiling changes in EV surface epitope expression in our rodent model of pregnancy with the aim of identifying a biomarker that could discriminate between the pregnant and non‐pregnant conditions.

**FIGURE 8:**
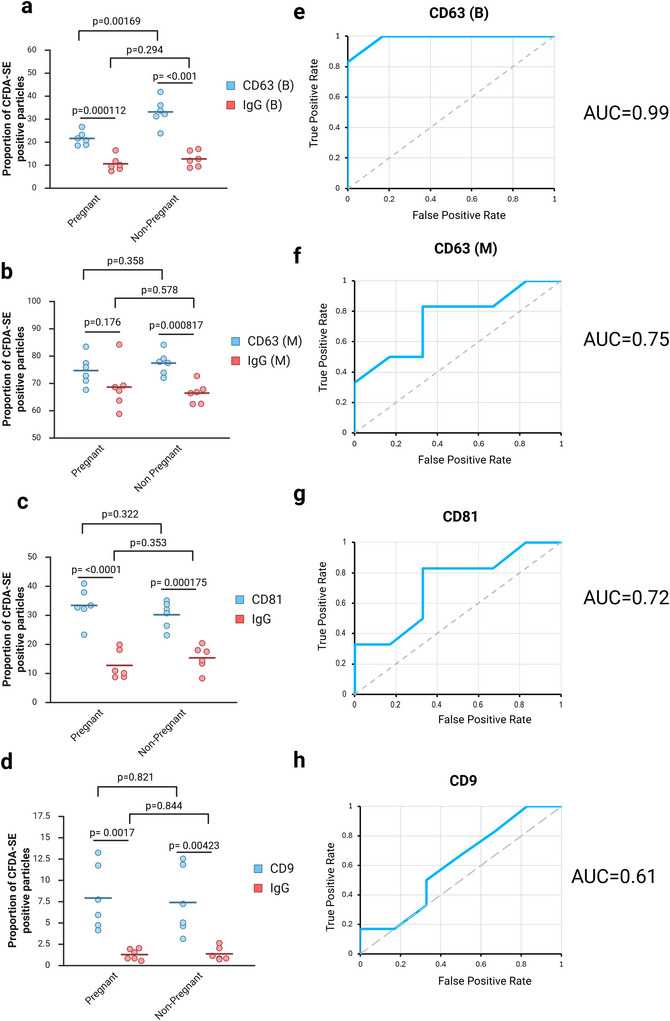
**Changes in tetraspanin display in plasma EVs during pregnancy**. (a–d) Flow cytometry data. Graphs to show proportion of CFDA‐SE positive particles expressing each biomarker. Each spot represents the mean of three technical replicates for one biological replicate, and the bars represent the group mean and standard deviation. *p* values indicate result of a *t* test. All samples are normally distributed (Shapiro–Wilk test) and of equal variance (Levene test). (e, f) ROC curves derived from the data shown in (a–d). (a, e) CD63 (B). (b, f) CD63 (M). (c, g) CD81. (d, h) CD9. CFDA‐SE, carboxyfluorescein diacetate succinimidyl ester; EV, extracellular vesicle; ROC, receiver operator characteristic.

The data revealed a significant reduction in the proportion of EVs expressing CD63 in the pregnant condition (21.5% ± 3.0%) relative to non‐pregnant controls using the CD63 (B) antibody (33.0% ± 5.9%; Figure 8a). Because this antibody contains a source of background, we repeated the assay using a second CD63 antibody, CD63 (M, Figure 8b). This antibody gave a much higher proportion of double‐labelled particles for all conditions (Figure 8b). We observed the same reduction in display for antibody CD63 (M) as for CD63 (B), but the difference was found to be not significant for the latter (Figure 8b). We observed no change in either CD81 (Figure 8c) or CD9 (Figure 8d).

To validate the specificity of these findings, we compared the proportion of EVs labelled within a given sample by each test antibody to its corresponding isotype control. As expected, we observed a highly significant difference for antibodies CD63 (B), CD81 and CD9. For example, the CD63 (B) antibody labelled 33.0% ± 5.9% of EVs within the non‐pregnant group while the corresponding isotype control labelled 12.6% ± 3.4% (Figure 8a). Furthermore, we did not observe a significant difference in the proportion of EVs labelled by an isotype control in the pregnant group compared to the non‐pregnant. These data suggest that the antibodies are resulting in specific EV labelling.

Antibody CD63 (M), in contrast, showed a difference between isotype and antibody only in the non‐pregnant group (Figure 8b). In the pregnant group (which has a lower mean signal), no difference was detected between the isotype and antibody indicating a lack of specificity.

To assess the usefulness of these antibodies as biomarkers to differentiate the pregnant from the non‐pregnant condition, we plotted ROC curves (Figure 8e–h). These data demonstrated that the CD63 (B) antibody is an excellent discriminator, with an AUC of 0.99 (Figure 8e), the CD63 (M) antibody performed less well, giving an AUC of 0.75 (Figure 8f). CD81 and CD9 gave AUC values of 0.72 and 0.61, respectively (Figure 8g, h).

## 4 Discussion

In this work, we have examined the fluorescent dye CFDA‐SE as a positive marker for detection of EVs of a size below the limit of detection of a standard flow cytometer. We then assayed these labelled EVs using fluorescent antibodies to detect EV surface tetraspanin display and tested the ability of this assay to discriminate between the pregnant and non‐pregnant conditions.

### 4.1 CFDA‐SE as a Positive EV Marker

CFDA‐SE fluorescence must be switched on through hydrolysis of the ester bond attaching the acetate groups and in theory this makes the dye non‐fluorescent until it is activated inside EVs. We observed a low level of fluorescence of the unbound dye in PBS, indicating a degree of non‐specific hydrolysis in solution (Figure 2d). We observed a significant increase in fluorescence in the presence of EVs, and this allowed us to define an EV as a particle with fluorescence above a threshold set at the level of the unbound dye in PBS (Figure 2b–d). Prior detergent treatment could reduce fluorescence to the latter level, indicating that fluorescence was induced in EVs (Figure 2d). One criticism of CFDA‐SE as a positive marker of EVs is its apparent vulnerability to false results due to swarm detection. Swarm detection refers to the phenomenon of multiple sub‐threshold particles being detected by the flow cytometer as a single particle, thereby giving a false reading of particle concentration (Buntsma et al. 2023). de Rond et al. (2018) reported swarm detection of CFDA‐SE labelled EVs in their analysis of unpurified plasma and in cell culture media. However, this problem appears to be limited to unpurified (and therefore protein‐rich) samples, and analysis of ultracentrifuge‐purified plasma indicates the absence of aggregates (Morales‐Kastresana et al. 2017). Our data (Figure 2e) suggest that swarm detection is not a problem in our assay. We observed that particle count estimated using CFDA‐SE positive particles was below that determined by NTA (Figure 6b). Particle size can be estimated by calibration of FSC‐H to values obtained using beads of known size, but our data suggest these values are significantly lower than those calculated by NTA (Figure 6c).

### 4.2 CFDA‐SE Specificity and False Positives

CFDA‐SE labelling has previously been used in EV flow cytometry analysis of a number of samples, including cell culture media (Morales‐Kastresana et al. 2017; De Rond et al. 2018; Pospichalova et al. 2015; Ender et al. 2020), plasma (Maia et al. 2020) and other body fluids (Pospichalova et al. 2015; Maia et al. 2020). EV‐enriched samples were prepared for these studies by ultracentrifugation (Morales‐Kastresana et al. 2017; Pospichalova et al. 2015; Ender et al. 2020), while SEC has not previously been used to prepare samples for CFDA‐SE labelling.

Our data indicate a specificity problem which has not been explored in detail in previous studies. We found that blood proteins such as albumin can also activate CFDA‐SE (Figure 3). This is in line with previous findings showing activation by BSA or complete culture media (De Rond et al. 2018; Ender et al. 2020). CFDA‐SE is able to react with proteins within cells because its succinimidyl group reacts with amino groups (Figure 1b) so it is logical that CFDA‐SE should also be able to bind to proteins such as albumin. The fluorescent signal most likely results from non‐esterase‐mediated degradation of CFDA‐SE to fluorescent CFSE, a process we have observed in our non‐EV samples (Figure 2d, PBS + CFDA‐SE). It is therefore important to identify any protein present in the sample that could lead to false positives. The two sources that we investigated were the EV SEC preparation itself and the staining reagents.

Plasma contains a large amount of protein, making it a more challenging sample to analyse than cell culture media, for example. For this reason, it is especially important to ensure that protein is removed during EV preparation. In one previous study, EVs in unpurified plasma were labelled with CFDA‐SE followed by an SEC cleanup step (Maia et al. 2020). Our data suggest this may result in false positives. We used the Exospin Mini commercial kit (concentration by precipitation followed by SEC). Precipitation is a non‐specific method that uses a water‐excluding polymer (proprietary in the Exopsin kit, but commonly polyethylene glycol) to precipitate EVs (Doyle and Wang 2019). Any contaminating proteins should also precipitate together with EVs. SEC is the key step that separates particles based on size. Published data testing the larger Exospin Midi column showed good separation between EVs and proteins (Welton et al. 2015). Our data support this. We found that SEC Fractions 1–4 (the first 180 µL) are enriched in EVs and largely lacking in protein (Figure 3). Further support for this assertion comes from the observation that detergent treatment is able to reduce the CFDA‐SE fluorescence to background levels (Figure 2d), suggesting lack of albumin, which would also activate CFDA‐SE.

Lipoproteins such as HDL and LDL are a possible contaminant, being of a similar size to EVs. TEM data revealing larger darkly stained particles suggest the possibility of such contamination (Figure 6e), but we cannot be certain of the identity of these particles. de Rond et al. (2018) reported that exogenous lipoproteins can activate CFDA‐SE, while others have made similar claims for other commonly‐used EV labels such as Annexin V and lactadherin immunostaining (Botha et al. 2022) and MemGlow (Brealey et al. 2024). Detergent treatment data argue against their presence in our samples.

A second source of albumin is the staining reagents used. BSA is commonly added to antibody storage buffers, and we found a good correlation between the presence of BSA in the buffer and activation of CFDA‐SE in the absence of EVs. This is problematic, especially when working in a species such as the rat where there are limited antibody choices. It is sometimes difficult to establish precisely what buffer antibodies are stored in as this information can be proprietary, and in the same of one of our antibodies (CD63 M) we were unable to obtain this information. Further optimisation could perhaps be achieved by using a buffer‐exchange column to remove BSA from antibody storage buffers before staining.

### 4.3 CFDA‐SE Sensitivity and False Negatives

False negatives could result from failure to label all EVs with CFDA‐SE or failure of the antibody to bind to all targets. Our findings that EV counts calculated by NTA are higher than those calculated by flow cytometry (Figure 6b) are in line with previous reports (Barnes et al. 2023). This may suggest either the presence of unstained EVs or the presence of non‐EV particles.

The results shown in Figure 7 indicates that the calculated proportion of EVs appearing to display a given antibody in our assay may be less than the true proportion. This seems to arise because the antibody is in limited supply and is not bound to all available sites. As the antibody concentration is increased, so the apparent proportion of double‐positive EVs increases. Thus, the assay can compare two populations stained in the same way, but cannot be used to draw conclusions regarding the relative proportions of different biomarkers.

### 4.4 Background Reduction

We tested two methods to increase the sensitivity and specificity of our assay: SEC cleanup (Figure 4) and trypan blue quenching (Figure 5).

We show that SEC is ineffective as a method to remove unbound dye (Figure 4). Our data show that the level of FL1 fluorescence following SEC purification is reduced to a value near that of buffer alone (Figure 4a), and we were unable to detect an FL4 signal, indicating the presence of antibody‐bound EVs in the output (Figure 4b). Our data further suggest that the movement of labelled EVs through the SEC column is impeded, resulting in elution of EVs largely in the same fractions as free dye (Figure 4c).

Morales‐Kastresana et al. (2017, 2020) have previously reported that SEC purification performed after CFDA‐SE staining successfully separates labelled EVs from hydrolysed (fluorescent) free dye, and that this is effective at increasing the sensitivity and specificity. This result would appear to be at odds with our own data. However, there are a number of important differences between our study and that of Morales‐Kastresana et al. (2017). Firstly, whereas Morales‐Kastresana et al. use buffer alone to set their baseline (their Figure 4a), we used the dye in buffer (Figure 1c). Thus, we are using a different baseline and have a different definition of background. In our assay, any spontaneously hydrolysed dye is excluded from the analysis by our threshold, and we are looking for an EV signal that is brighter than a single molecule of CFDA‐SE, making the assumption that each EV will carry multiple CFDA‐SE molecules. Secondly, there are a number of differences in the EV sample under investigation. Importantly, Morales‐Kastresana et al. analysed CFDA‐SE labelled EVs while we used CFDA‐SE and antibody labelled EVs. It is possible that the presence of antibodies on the EV surface impeded passage through the column. Further, Morales‐Kastresana et al. analysed EVs from serum‐depleted cell culture medium, which is a much simpler substrate than the plasma used in our assay. They purify EVs initially by ultracentrifugation rather than by SEC, the former may alter the morphology of the EVs, making them more likely to pass unimpeded through the column. Finally, there are differences in the SEC columns used: the Exospin column we used has a pore size of 30 nm while Morales‐Kastresana et al. used a Cytiva NAP‐5 column containing Sephadex G25 (pore size 25 nm). The smaller pore size of the latter may promote EV recovery. Thus, a direct comparison of the two results is impossible. Although this may yet prove to be a useful method, caution must be used in purifying EVs post‐staining and further work is needed to determine the optimal pore size and resin type for this method and to investigate the influence of processing steps on EV ‘stickiness’ within the column.

Although trypan blue is a useful tool to quench extracellular fluorescence in cell biology assays due to its exclusion from live cells, our data suggest that it is unsuitable for quenching CFDA‐SE background signal in EV assays (Figure 5). The data suggest that trypan blue quenches FL1 fluorescence within the EV along with any external background fluorescence (Figure 5a). This suggests that in this case the EV membrane is behaving like the membrane of a dead cell and failing to exclude charged molecules. Furthermore, trypan blue treatment results in strong background FL4 fluorescence (Figure 5b), which is likely to mask any FL4 antibody signal. This result is in line with previous findings suggesting that trypan blue binds BSA and other proteins non‐specifically resulting in an increase in its red fluorescence emission (Kerschbaum et al. 2021; Shilova et al. 2017; Avelar‐Freitas et al. 2014).

### 4.5 Validity and Interpretation of the Biomarker Assay

We tested the ability of our assay to act as a binary discriminator to detect a difference between two conditions, such as might be useful in a prenatal diagnostics test. In our assay we asked whether EV tetraspanin display levels could discriminate between the pregnant and non‐pregnant conditions.

The CD63 (B) antibody revealed a highly significant reduction in CD63 in the pregnant condition (Figure 8a), and this proved to be a good discriminator of the two conditions, with an AUC of 0.99 (Figure 8e). The optimal threshold gave a sensitivity of 1 and a specificity of 0.83. Interestingly, a second antibody – CD63 (M) – detecting the same biomarker performed less well. It failed to detect a significant difference (Figure 8b) and was a less good discriminator (AUC = 0.75, sensitivity = 0.83, specificity 0.67 at optimal threshold; Figure 8f). The high readout for this assay relative to CD63 (B) suggests a higher false positive level, reducing the signal:noise ratio and making it more difficult to detect a difference. The composition of the CD63 (M) storage buffer is proprietary. That a difference between the conditions was present in the latter assay is suggested by the comparison to the isotype control, which was only significantly different in the non‐pregnant condition.

CD81 and CD9 do not appear to be suitable biomarkers in this assay: CD81 gave an AUC of 0.72 and CD9 0.61. Both antibodies are supplied in a BSA‐free storage buffer and therefore false positives would not be expected to be a problem in these assays.

It is clear that the readout of the assay does not reflect the true proportion of EVs expressing a given biomarker, but must be treated as an arbitrary unit unique to each assay. For this reason, we cannot compare results between different biomarkers because the composition of the antibody storage buffers as well as variables such as antibody concentration may vary and influence the readout.

We show that BSA present in the antibody storage buffer is a source of background. However, because the background is coming from a reagent added to each sample, and not from a property of the sample itself, this should not influence pairwise comparisons between two samples treated in the same way. The presence of such background will influence the signal:noise ratio and perhaps make it more difficult to see a difference and therefore taking steps to reduce this, such as choosing BSA‐free antibodies, would be beneficial. Of greater importance is to obtain test samples free of impurities, which may cause variation between groups. As discussed above, SEC purification appears suitable for this.

Thus, the assay is suitable for diagnostic applications in which simply the existence of difference between conditions is key.

### 4.6 Biological Significance of Observed Changes in EV Expression During Pregnancy

A recent systematic review of 152 clinical studies reported an overall increase in EV concentration in pregnancy, although the study revealed wide variations in reported results (Barnes et al. 2023). We observed a 1.9‐fold increase in the pregnant condition, but this was below the threshold of significance due to high variance. It is possible that a larger sample size could reveal a small increase.

Our data suggest a specific reduction in CD63‐positive EVs in pregnancy. It is interesting to note that this change in CD63 was not matched by a detectable change in CD9 or CD81 and was not accompanied by any change in particle size distribution. Although a small increase in CD81 was observed in the pregnant condition, this was not significant. Small EVs may be broadly divided into those derived from the endosomal pathway (exosomes) and those derived by budding at the cell surface from plasma membrane (ectosomes). Although all three tetraspanins may be expressed in any EV subtype, CD63 is generally regarded as a marker of the endosomal‐derived exosome class of small EVs, whilst CD9 and CD81 are more commonly localised to the plasma membrane (Mathieu et al. 2021; Fordjour et al. 2022). Analysis of single vesicles in human amniotic fluid by super resolution microscopy indicates that most EVs are either triple positive for these tetraspanins or are double positive for CD9 and CD81 (Gebara et al. 2022). Han et al. (2021) analysed expression in single vesicles across a range of cell lines. Although they found variations between cell types, they also generally observed co‐expression of CD9 and CD81. Therefore, CD63 does appear to differentiate between classes of small EVs (triple positive against CD9/CD81 positive), and these populations may represent ectosomes and exosomes. Thus, it is possible that we are observing in rats the result of a switch between from plasma membrane derived budding of ectosomes to endosomal production of exosomes during pregnancy.

It is difficult to say whether this apparent shift reflects a physiological change occurring during pregnancy. We did not investigate the source of these EVs and cannot say whether this represents signalling derived from the placenta or whether it instead reflects a change in maternal physiology in response to pregnancy. Pregnancy is associated with a dampening of the immune system to permit tolerance of the foetus by the mother, and it is thought that EVs released by the placenta play a role in this process. For example, in man placental EVs carry the immunosuppressant HLA‐G (Kshirsagar et al. 2012), and this has been proposed to mediate this function (Rebmann et al. 2016).

Although the rat is sometimes used to investigate placental EVs derived from clinical or human cell samples (Dutta et al. 2020), little is known of the nature of endogenous placental EVs in the rat. There are major differences in placental anatomy and function between species, and this makes the use of animal models problematic (Block et al. 2021). Many of the well know placental EV markers, such as HLA‐G and the miRNA C19MC cluster, were identified in human EVs and have been found to be primate specific (Adamova 2023). It is unclear whether another human placental EV marker, PLAP, is placental specific in rats (Adamova 2023; Avelar‐Freitas et al. 2014). This highlights the need for further study of the rat if it is to be used to model changes in human pregnancy.

### 4.7 EVs as Clinically Useful Biomarkers

In this work, we explored the validity of EVs in detecting a binary difference between two conditions. However, unlike static biomarkers such as DNA variation, EVs potentially provide a real‐time, dynamic readout of health that goes well beyond a simple healthy/unhealthy readout, offering the potential for monitoring of prognosis or of a more sophisticated stratification of disease subtypes (Mohapatra et al. 2026; Bernáth‐Nagy et al. 2024). We have here focussed on a difference in display of a biomarker common to many EVs, but future work may wish to focus on more sophisticated EV surface biomarkers which provide information on EV origin and targeting, for example, while analysis of the miRNA cargo provides a more detailed picture of EV signalling. As we show here, these assays rely on careful consideration of sources of error within the methodology and standardisation of procedures will be critical to development of clinical tests (Ayers et al. 2019). The challenge in the future will be to find a way to read this complex information using a simple technology translatable to clinical practise.

## 5 Conclusions

We have demonstrated that a binary difference in EV tetraspanin display may be detected using a liquid biopsy of maternal blood using CFDA‐SE and antibody labelling with a basic flow cytometry setup. These results suggest that the method could be applied to clinical samples and potentially could be useful in the diagnosis of congenital disease.

## Funding

This work was funded by an LJMU Institute of Health Research cross‐faculty PhD studentship (P.A., I.M.D., A.K.P.), an LJMU Vice Chancellor's PhD studentship (L.S., I.M.D., A.K.P.), funding from Heart Research UK (I.M.D., A.K.P.) and LJMU seedcorn funding (I.M.D.). Rosetrees Trust Pilot/Seed Corn and IBCarb Network in Industrial Biotechnology Proof of Concept grant funding (A.K.P.) assisted in conceiving the CFDA‐SE labelling approach. A small bursary from LJMU Institute of Health Research (I.M.D.) funded additional reagents required for revisions.

## Ethics Statement

The protocol for animal work described in this study was reviewed and approved by the Liverpool John Moores University Animal Welfare and Ethics Review Board in January 2020 (Ref: ID_PA/2023‐3).

## Consent

Not applicable.

## Permission to Reproduce Material From Other Sources

Not applicable.

## Clinical Trial Registration

Not applicable.

## Conflicts of Interest

The authors declare no conflicts of interest.

## Supporting information



Supporting Information: jex270145‐sup‐0001‐SuppMat.docx

## Data Availability

The data that support the findings of this study are available from the corresponding author upon reasonable request.
