# Supplementary material for: Flow Cytometric Detection of Biomarker Changes in CFDA‐SE‐Labelled Plasma Extracellular Vesicles Using a Rodent Pregnancy Model of Prenatal Diagnostics
Source: J Extracell Biol. 2026 May 5;5(5):e70145. doi: 10.1002/jex2.70145 (PMC13145351; doi:10.1002/jex2.70145)
Supplement: Supplementary file 1 — Supporting Information: jex270145‐sup‐0001‐SuppMat.docx [file JEX2-5-e70145-s001.docx]

**SUPPLEMENTAL DATA**


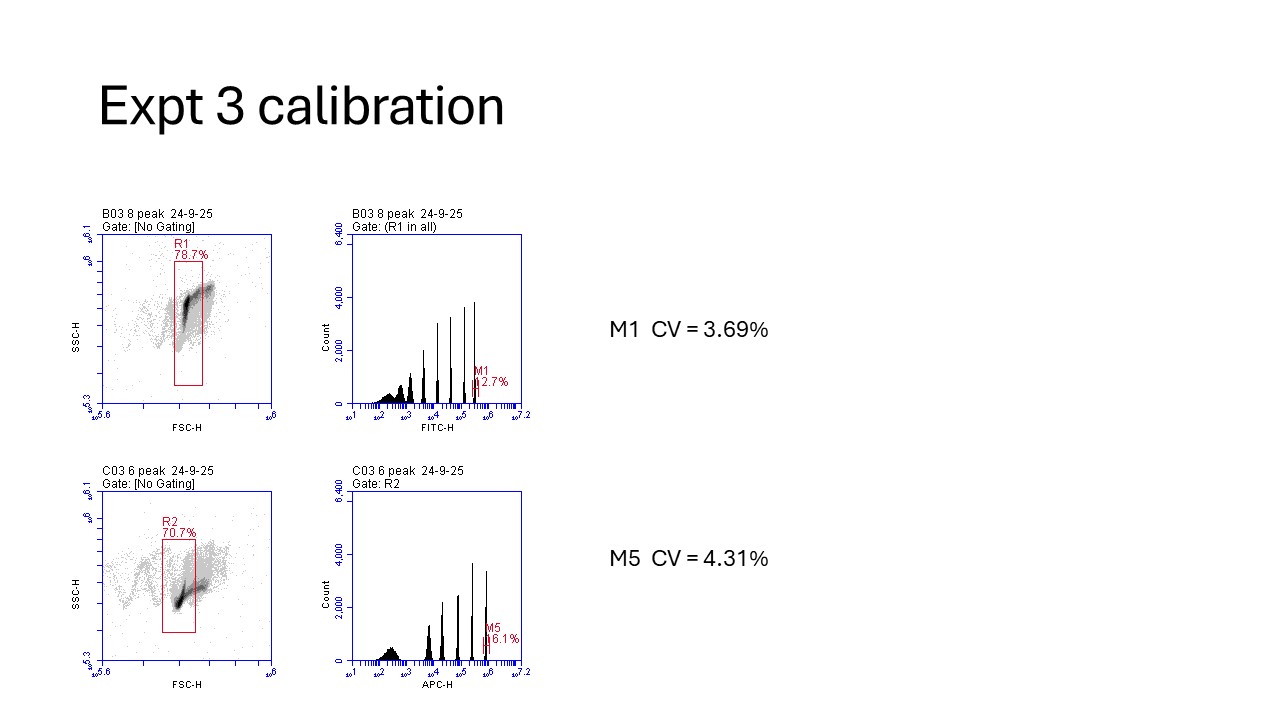


**Supplementary Figure 1. Calibration data for the BD Accuri C6+**


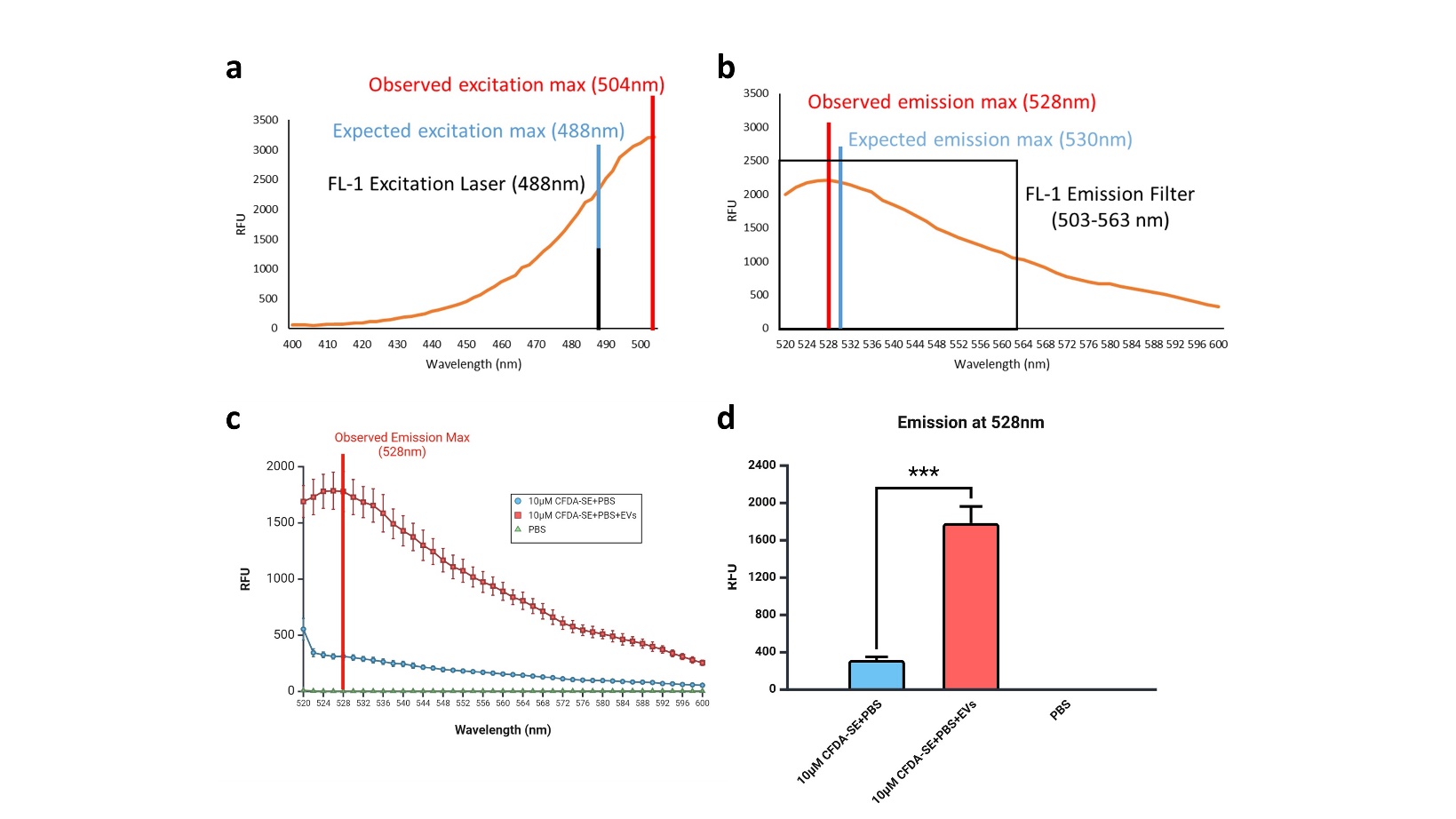


**Supplementary Figure 2. CFDA-SE fluorescence is detected with the FL1 channel and activated by EVs**

Plate reader data. **a**. Excitation scan. A 19µM solution of CFDA-SE in PBS was analysed. Emission at 545nm is shown for the excitation range 400-504nm. The observed excitation maximum (504nm, red line) was higher than the published maximum (488nm, blue line). **b**. Emission scan. A 19µM solution of CFDA-SE in PBS was analysed. The fluorophore was excited at 480nm and emission is shown for the range of 520-600nm. The observed emission maximum (528nm, red line) was close to the published maximum (530nm, blue line). **c**. Emission scan of CFDA-SE in the presence or absence of EVs. The fluorophore was excited at 488nm and emission is shown for the range of 520-600nm. The graph shows the mean plus standard deviation of three technical replicates. **d**. Emission at 528nm. Data is derived from the graph in c. Graph shows mean plus standard deviation of three technical replicates. There is a significant increase in fluorescence intensity in the presence of EVs (p= 0.000149, independent samples two tailed t-test. Data normally distributed (Shapiro-Wilk test) and of equal variance (Levene’s test). RFU = relative fluorescence units.


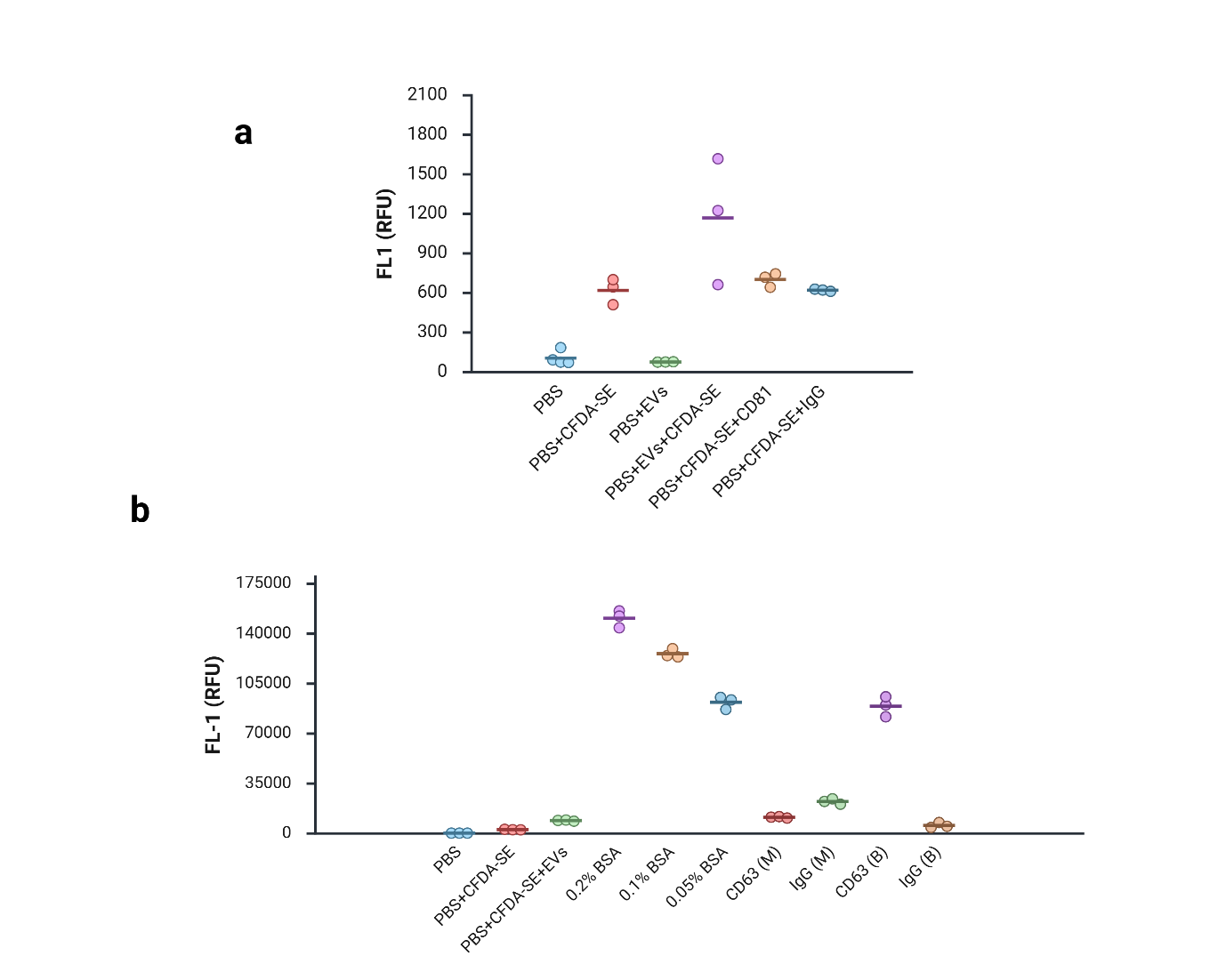


**Supplementary Figure 3. Further antibody controls**

Plate reader data

a. The CD81 antibody does not activate CFDA-SE in the absence of EVs.

b. Comparison of the Biolegend CD63 antibody (CD63 B) to the Miltenyi CD63 antibody (CD63 M). CD63 (B) shows strong activation of CFDA-SE in the absence of EVs to a level comparable to a 0.05% solution of BSA. CD63 (M) shows much reduced activation.


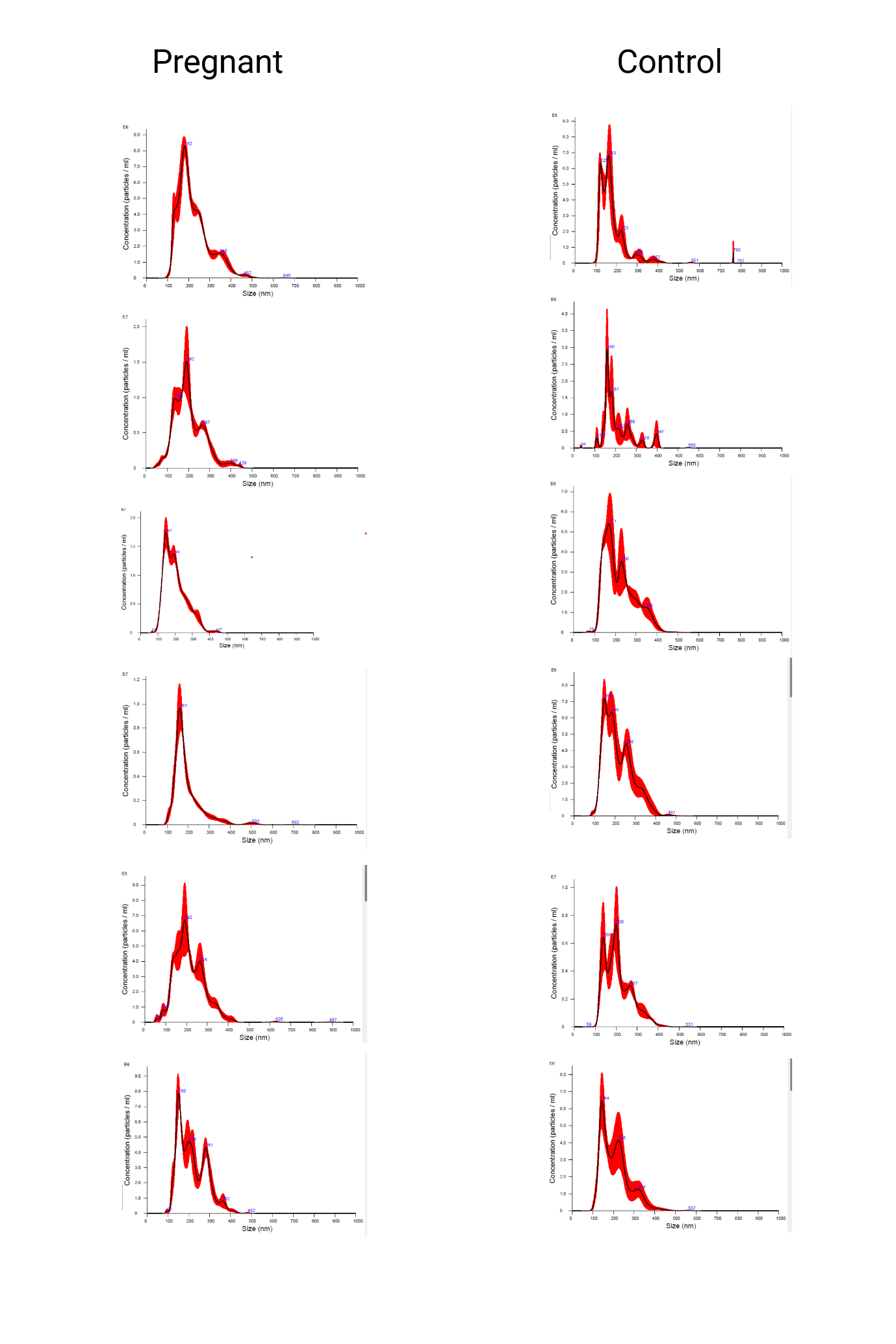


**Supplemental Figure 4. Nanoparticle tracking data.**

Figure shows data from all 12 rats used in the analysis.


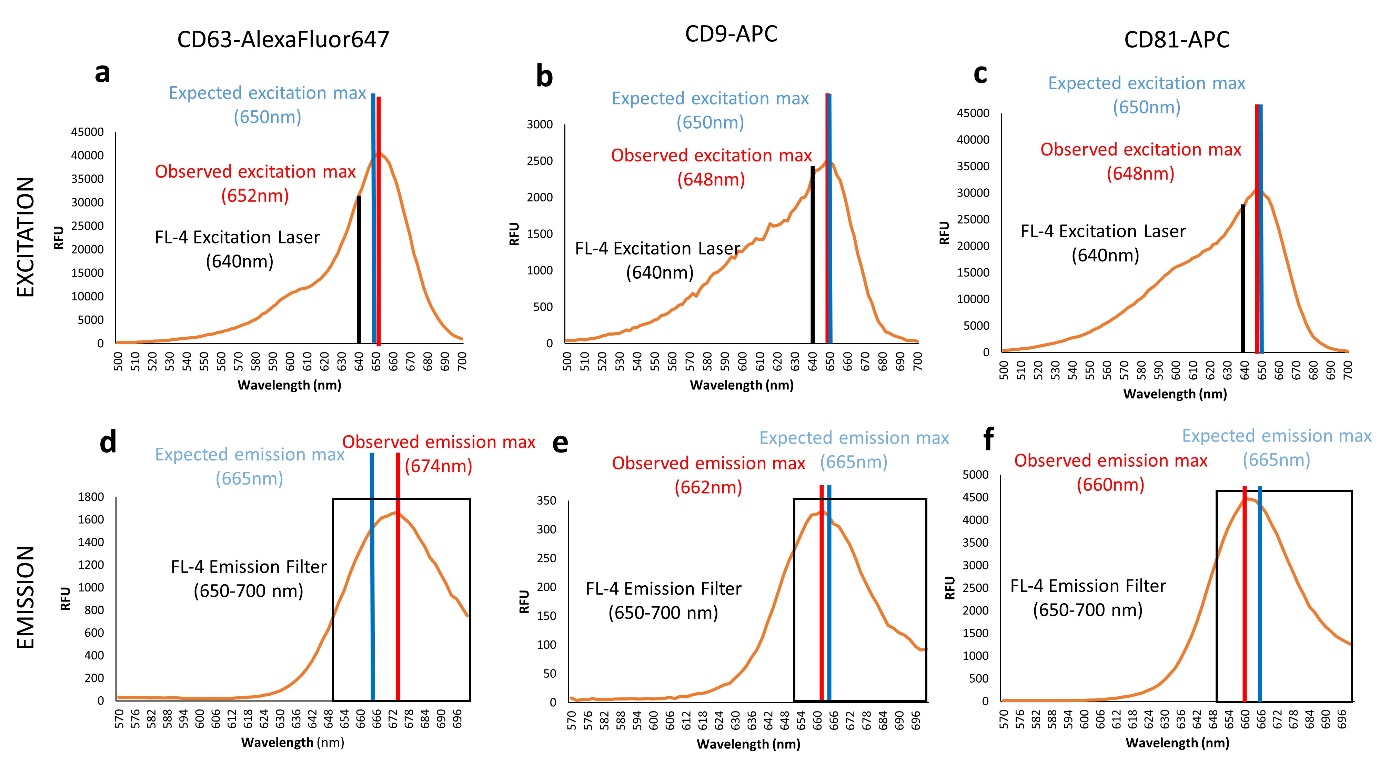


**Supplementary Fig 5. Characterisation of the fluorescent antibodies used in this study.**

Plate reader data showing activity of unbound antibody in PBS. Three antibodies were tested: anti CD63 AlexaFluor 647 (a, d), anti CD9 APC (b, e) and anti CD81 APC (c, f)**. a-c**. Excitation scans across the 550-700nm range. The observed excitation maxima (red lines) was within 2nm of the expected (blue lines) for all fluorophores. (A) CD63-AlexaFluor647; the recorded experimental value was 652nm. **d-f.** Emission scans. Observed emission maxima (red lines) varied by 3-9nm from expected (blue lines), but in all cases was within the range picked up by the FL-4 filter (black box).

a.

|  | **Antibody** | **Catalogue** | **Storage Buffer** |
| --- | --- | --- | --- |
| **Primary** | CD81 | Biolegend  104910 | PBS, pH 7.2, 0.09% sodium azide. |
|  | CD9 | Biolegend  206504 | PBS, pH 7.2, 0.09% sodium azide. |
|  | CD63-B | Biorad MCAA4754A647 | PBS, 0.09% sodium azide, 1% BSA |
|  | CD63-M | Miltenyi  130-134-122 | Buffer, 0.05% sodium azide, unspecified proprietary protein stabiliser |
| **Secondary** | Hamster IgG1-k APC | Biolegend  400912 | PBS, pH 7.2, 0.09% sodium azide. |
|  | Mouse IgG1-k  APC | Biolegend  400120 | PBS, pH 7.2, 0.09% sodium azide. |
|  | Mouse IgG1  AlexaFluor 647 | eBioscience  S1-4714-81 | PBS, pH 7.2, 0.09% sodium azide. |
|  | Human recombinant  IgG1 APC | Miltenyi  130-113-446 | Buffer, 0.05% sodium azide, unspecified proprietary protein stabiliser |

**Supplemental Table 1. Composition of storage buffers for antibodies used in this study**
